# SMG-1 and mTORC1 Act Antagonistically to Regulate Response to Injury and Growth in Planarians

**DOI:** 10.1371/journal.pgen.1002619

**Published:** 2012-03-29

**Authors:** Cristina González-Estévez, Daniel A. Felix, Matthew D. Smith, Jordi Paps, Simon J. Morley, Victoria James, Tyson V. Sharp, A. Aziz Aboobaker

**Affiliations:** 1 Centre for Genetics and Genomics, University of Nottingham, Queens Medical Centre, Nottingham, United Kingdom; 2 Department of Zoology, University of Oxford, Oxford, United Kingdom; 3 Department of Biochemistry, School of Life Sciences, University of Sussex, Brighton, United Kingdom; 4 School of Biomedical Sciences, University of Nottingham Medical School, Nottingham, United Kingdom; Massachusetts Institute of Technology, United States of America

## Abstract

Planarian flatworms are able to both regenerate their whole bodies and continuously adapt their size to nutrient status. Tight control of stem cell proliferation and differentiation during these processes is the key feature of planarian biology. Here we show that the planarian homolog of the phosphoinositide 3-kinase-related kinase (PIKK) family member *SMG-1* and mTOR complex 1 components are required for this tight control. Loss of *smg-1* results in a hyper-responsiveness to injury and growth and the formation of regenerative blastemas that remain undifferentiated and that lead to lethal ectopic outgrowths. Invasive stem cell hyper-proliferation, hyperplasia, hypertrophy, and differentiation defects are hallmarks of this uncontrolled growth. These data imply a previously unappreciated and novel physiological function for this PIKK family member. In contrast we found that planarian members of the mTOR complex 1, *tor* and *raptor*, are required for the initial response to injury and blastema formation. Double *smg-1* RNAi experiments with *tor* or *raptor* show that abnormal growth requires mTOR signalling. We also found that the macrolide rapamycin, a natural compound inhibitor of mTORC1, is able to increase the survival rate of *smg-1* RNAi animals by decreasing cell proliferation. Our findings support a model where *Smg-1* acts as a novel regulator of both the response to injury and growth control mechanisms. Our data suggest the possibility that this may be by suppressing mTOR signalling. Characterisation of both the planarian mTORC1 signalling components and another PIKK family member as key regulators of regeneration and growth will influence future work on regeneration, growth control, and the development of anti-cancer therapies that target mTOR signalling.

## Author Summary

Planarian flatworms have a remarkable ability to regenerate that has driven the curiosity of scientists for more than a century. They are also able to continuously grow or degrow their bodies, depending on food availability. Around 25% of the cells in the planarian body are adult stem cells, which are responsible for this incredible plasticity. The initial response of planarians to injury is characterised by a rapid increase in stem cell division. Subsequently planarians form a specialised new tissue called the regenerative blastema to replace missing tissues. Currently, very little is known about the molecular signals controlling the response to injury or the tight regulation of growth. Here we discovered that a gene called *Smg-1* and the conserved mTOR signalling pathway, a central regulator of animal growth, are both regulators of this process. SMG-1 is required to limit and act as a brake on the initial response to injury and ensure that it does not run out of control, while in contrast mTOR signalling is required to drive this process forward. Loss of SMG-1 leads to hyperactive responses to injury and subsequent growth that continues out of control. Eventually, these animals form outgrowths, which display several hallmarks of human cancers. These opposing roles suggested that *Smg-1* phenotype would require mTOR signalling, and by reducing mTOR signalling and SMG-1 activity at the same time we found that this was the case. We conclude that *Smg-1* is a novel regulator of regeneration and animal growth with an antagonistic role to mTOR signalling in planarians.

## Introduction

Planarian flatworms have a remarkable plasticity that has driven the curiosity of scientists for more than a century [1], [2]. These abilities rely on adult stem cells called neoblasts, which are able to give rise to all types of differentiated cells in the planarian body [3], [4]. Planarians have a growing importance as a model system as one of the more extreme examples of regeneration [5]–[7]; even tiny fragments of their body are able to regenerate a completely proportioned organism in about 2 weeks. After amputation neoblasts undergo a broadly distributed increased mitotic response to injury in the first 4–10 h and a second more spatially restricted mitotic response at 48–72 h, specifically in response to missing tissue [8], [9]. Neoblast progeny migrate and form an unpigmented tissue called the blastema. The blastema becomes progressively pigmented and neoblasts terminally differentiate to form missing structures. In addition to the formation of new structures at the blastema, homeostatic changes in the old tissue are also necessary for the planarian to remodel its body. Similarly, uninjured planarians constantly replace aged differentiated cells from the mitotic progeny of neoblasts [10]. The extent and duration of mitotic responses, neoblast migration, the differentiation of neoblast progeny and mechanisms to report successful regenerative outcomes that ultimately down regulate growth responses must exist through the whole animal. Exquisite control of these processes is necessary, as failure would lead to aberrant/incomplete regeneration or conversely outgrowths that disturb normal physiology. Recent insights from careful observation of degrowth processes has shown that the numbers of neoblast progeny are decreased and cell death increased in response to nutrient shortage [11]. This response, as in other animals, requires insulin signalling [12]. It seems more than likely that other signalling systems regulating these processes will be conserved across animals. The extreme nature of planarian life history traits mean that it is a simple and accessible system to identify new regulators of this process which may prove to be evolutionarily conserved.

Here we demonstrate this potential by identifying the PIKK family member SMG-1 as a key regulator of planarian growth. Its loss results in a deregulation of planarian growth leading to lethal ectopic outgrowths as the stem cell response to injury runs out of control. We also identify the central components of mTOR signalling, arguably the nexus for signals controlling growth across animals and show that the mTORC1 complex is necessary for blastema formation and growth. We find that the novel physiological function for SMG-1 acts antagonistically with mTORC1 signalling. Our findings have broad implications for understanding regeneration, growth and cancer.

## Results

### *Smed-smg-1* restricts the injury response and blastema growth by regulating neoblast proliferation

**Figure 1:**
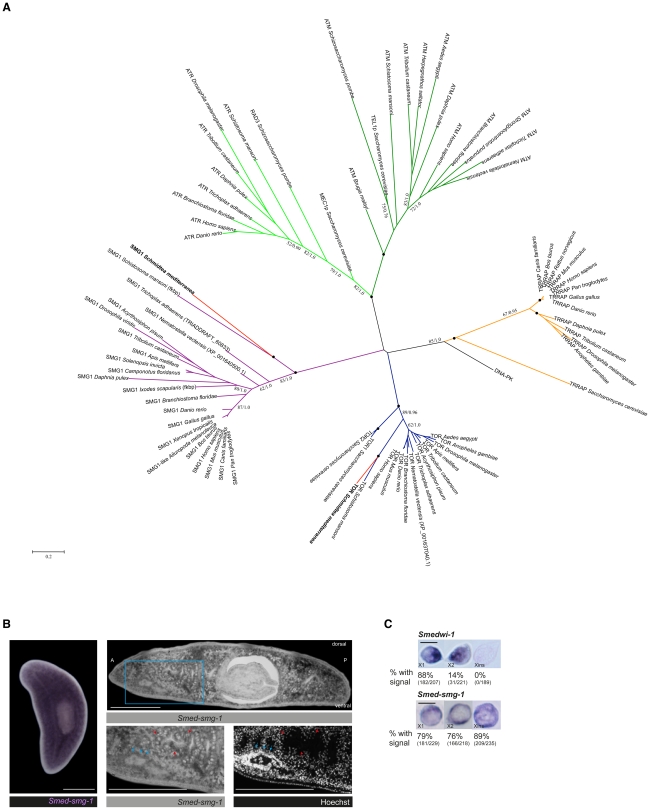
*Smed-smg-1* is a bona fide *SMG-1* and is broadly expressed in the whole planarian body. A. Maximum Likelihood phylogenetic tree of PIKK full-length proteins. Phylogeny inferred with RAxML (GTR+Γ+I), numbers correspond respectively to Bootstrap supports and Bayesian inference Posterior Probabilities. A black dot indicates a clade with Bootstrap support superior to 95% and Bayesian Posterior Probabilities (PP) values of 1,0. Values under 50% or 0.5 are not indicated. The scale bar indicates the number of changes per site. For accession numbers corresponding to each terminal see Table S1. B. *Smed-smg-1* is broadly expressed in the planarian body (n = 30/30 in three independent experiments). The right panels show an *in situ* hybridization on 10 µm paraffin sections where the staining is shown in black-dark grey. High magnifications show expression in gastrodermal cells (red arrows) and neurons of the brain ganglia (blue arrows). A, anterior; P, posterior. C. ISH on FACS-isolated cells for the three cell populations already described based on sensitivity to irradiation [71]. X1 and X2 are irradiation sensitive cells and thus contain neoblasts, while Xins is insensitive and thus formed by differentiated cells. Representative results are shown. Percentages are signal-positive cells. *Smedwi-1* was used as a positive control and neoblast marker. *Smedwi-1* is mostly expressed in the X1 population [72]. *Smed-smg-1* is expressed in all three populations. Scale bars indicate 300 µm and 10 µm for single cell images.

In an RNAi screen attempting to identify novel signals that regulate correct growth in *Schmidtea mediterranea* we identified a gene with high identity to human *SMG-1* (*hSMG-1*, suppressor with morphogenetic effect on genitalia). Phylogenetic and conserved domains analyses supported the hypothesis that this gene is a homolog of hSMG-1 leading us to call it *Smed-smg-1* (Figure 1A, Figures S1 and S2; GenBank Accession Number JF894292). We found that *Smed-smg-1* is very broadly expressed through all planarian tissues, including neoblasts (Figure 1B and 1C). SMG-1 is the most recently described member of the phosphoinositide 3-kinase-related kinase family (PIKKs) [13], [14]. An SMG-1 homolog in *C. elegans* has been described as having a role in the RNA surveillance nonsense-mediated mRNA decay (NMD) pathway [15]. SMG-1 loss also results in lifespan extension in *C. elegans*, through an activity unrelated to its role in NMD [16]. Like other PIKKs, hSMG-1 is implicated in genome surveillance in human cells responding to stress. For example hSMG-1 is activated by DNA damage [17], during tumour necrosis factor-α-induced stress [18], cell cycle checkpoint signalling under oxidative stress [19] and negatively regulates HIF-1α activity in hypoxia [20]. Despite the fact that hSMG-1-depleted human cells display an increased level of spontaneous DNA damage [17] the physiological roles of SMG-1 in the absence of genotoxic stress have not been further studied.

**Figure 2:**
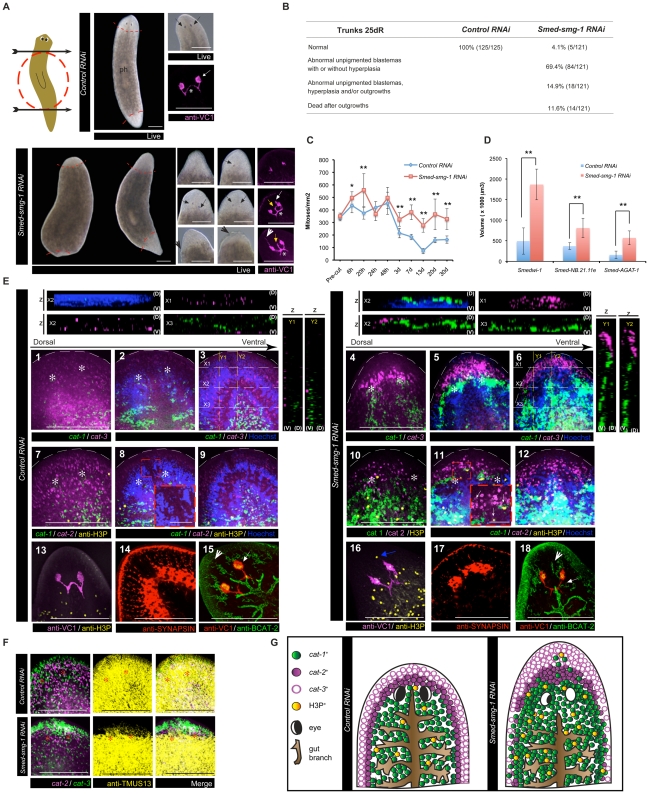
*Smed-smg-1* is required to restrict blastema growth during regeneration. A. The cartoon shows the levels of transverse amputation performed in the experiment (arrows) and highlights the trunk piece (dotted red circle), which was kept to follow anterior and posterior regeneration in all the experiments. Panels show 20–25 dR trunks. Dotted red lines define the blastemas. ph indicates the pharynx. *Smed-smg-1(RNAi)* animals show varying size of abnormal unpigmented blastemas with several degrees of eye differentiation displayed by the eye pigmentary cup in the live images (black arrows) and the VC1 staining. Asterisk shows a thickened optic chiasm, the white arrow shows an extra eye as an accumulation of unilateral photoreceptor clusters, always more anteriorly positioned than the original eyes, the yellow arrow shows abnormal axonal projections, and the white arrowhead shows an aberrant antero-dorsal photoreceptor projection. The black arrowhead shows epidermal hyperplasia. B. Table summarising the phenotypes observed in trunks at 25 dR; hyperplasia refers to epidermal hyperplasia observed in live animals. C, D. Mitotic numbers during different time points of regeneration and volume of the different neoblast compartments at 20 dR, respectively. Error bars are s.d from the mean and asterisks indicate P<0.05 (one asterisk) or P<0.01 (two asterisks) using two-tailed Student's test with equal sample variance. C. n≥5 planarians per time point in at least two independent experiments; D. n≥4. E. Panels show maximum confocal projections for the dorsal-most sections (1, 7, 4 and 10), the medial sections (2, 8, 5 and 11) and the ventral-most sections (3, 9, 6 and 12) of 20 d anterior regenerating trunks. Asterisks indicate the position of the eyes. Panels show the distribution of neoblasts (*cat-1*), neoblast early progeny (*cat-2*) and neoblast late progeny (*cat-3*) markers in controls versus *Smed-smg-1(RNAi)* animals. The inset in 8 and 11 show the presence of *cat-1* and H3P^+^ cells in the *cat-2* and *cat-3* compartment in front of the eyes in *Smed-smg-1(RNAi)* animals. Strips represent confocal XZ or YZ projections (D is dorsal and V is ventral) demonstrating a greater volume of neoblasts and progeny, an accumulation of *cat-1* cells dorsal to the brain ganglia (X2 and Y1) and a higher accumulation of *cat-3* at the anterior tip of the planarian (X1, Y1, Y2) in *Smed-smg-1(RNAi)* compared to controls. n≥4. Lower panels show H3P^+^ cells (panels 13 and 16, blue arrow) in front of the eyes (maximum confocal projection; n = 4/8), a very undeveloped brain labelled by anti-SYNAPSIN staining (panels 14 and 17; dorsal maximum confocal projection), an anterior gut branch (seen in BCAT-2 staining; maximum projection) aberrantly located with respect to the position of the eyes (n = 4/6, the white arrow indicates the most anterior level reached by the anterior gut branch) and the epithelium of protonephridial tubules stained by anti-BCAT-2 not reaching the tip of the planarian (panels 15 and 18; white arrow head; n = 6/6) in *Smed-smg-1(RNAi)* animals. F. Panels show maximum confocal projections of 20 d anterior regenerating trunks. The tip of the blastema shows minimal differentiation of muscle cells (stained with anti-TMUS13) in *Smed-smg-1(RNAi)* planarians. n = 3/3. G. Cartoons summarising the phenotype observed during anterior regeneration of 25 dR trunks. Scale bars represent 300 µm. Scale bars in (E) high magnification images represent 50 µm.

Planarians were injected with *Smed-smg-1* dsRNA and amputated in order to observe regeneration (Figure 2A). *gfp* dsRNA, a sequence not present in *S. mediterranea* genome, was used as control for all RNAi experiments. *Smed-smg-1(RNAi)* induced regeneration phenotypes became apparent in live animals from 7 days of regeneration (7 dR) when blastemas displayed minimal differentiation (n = 121/121) compared to controls (Figure S3). At 25 dR, when controls had completed regeneration, ∼70% of *Smed-smg-1(RNAi)* planarians still had unpigmented blastemas of varying size sometimes with apparent epidermal hyperplasia (Figure 2A, 2B), and ∼27% either displayed outgrowths/hyperplasia or had already died after forming outgrowths (Figure 2A, 2B). Eventually, all planarians that had abnormal blastemas progressed to form outgrowths and died (30–38 dR; 116/121) (Figure S4). The observation of hyperplasia and outgrowths suggested that *Smed-smg-1* might regulate neoblast proliferation. We checked the pattern of mitotic neoblasts at different time points during regeneration by using the Histone H3 phosphorylated at serine 10 (anti-H3P) antibody [21] (Figure 2C). We observed that *Smed-smg-1(RNAi)* planarians had a hyper-proliferative pattern of neoblast division with a higher (P<0.05) and clearly extended (P<0.01) 6 hR mitotic peak in response to initial injury and higher baseline levels of proliferation from 3 dR that fail to return down to normal levels (P<0.01). Planarians were never depleted for neoblasts (Figure 2D) and showed higher levels of proliferation than controls even when death was imminent at 30 dR (Figure 2B). To understand whether the higher proliferative response to amputation in *Smed-smg-1* RNAi animals was due to an increasing number of neoblasts present before amputation, we quantified the number of anti-H3P positive cells and the number of neoblasts positive for *Smedwi-1*, a marker for neoblasts [22], before amputation. We observed similar numbers of neoblasts (P>0.05) suggesting that differences in proliferation and neoblast number become apparent after amputation (Figure 2C and Figure S5). Our data suggest that *Smed-smg-1(RNAi)* results in a hyper-proliferative response after injury/amputation and that this eventually results in lethal outgrowths. Given that *Smed-smg-1(RNAi)* led to the formation of unpigmented blastemas, suggesting a lack of terminal differentiation, we next wished to assess the detailed dynamics of neoblasts and their progeny in relation to this phenotype.

### *Smed-smg-1(RNAi)* blastema growth is characterised by an uncontrolled accumulation of cycling neoblasts and their progeny with differentiation defects

Given the observation that *Smed-smg-1(RNAi)* animals showed unpigmented blastemas at 20–25 dR (Figure 2A), we next assessed the ability of neoblasts to differentiate. We used markers expressed in neoblasts, recent neoblast progeny or older neoblast progeny. Consistent with the study that originally defined these markers [23], we could observe increasingly peripheral expression domains for the different markers of these three cellular compartments in 20 dR control animals. *Smedwi-1* (neoblast marker) is expressed deeper in the body; *Smed-NB.21.11e* (early post mitotic progeny) is expressed peripherally to *Smedwi-1* and *Smed-AGAT-1* (late post mitotic progeny), which is the most peripherally expressed (Figure 2E). In *Smed-smg-1(RNAi)* animals we observed an antero-dorsal expansion of the *Smedwi-1* ^+^ neoblast compartment at anterior-facing blastemas and posterior-dorsal expansion at posterior-facing blastemas as early as 7 dR (Figure S3) that was even more pronounced at 20 dR (Figure 2E1–E12, Figure S6 and Figure S7). This expansion clearly encroached on the early and late progeny compartments (Figure 2E, Videos S1 and S2). This expansion was corroborated by the presence of a large front of cycling neoblasts (*Smedwi-1* ^+^ cells and H3P^+^ cells) present around the eyes that also reached anterior to the eyes, a region which is usually devoid of cycling neoblasts in normal worms [24] (Figure 2E2, E5, E8, E11, E13, E16) and by quantifying the number of *Smedwi-1* ^+^ from the region of the eyes until the tip of the head (P<0.001; Figure S6). Furthermore, the early and late progeny compartments had also higher proportional volumes than controls (Figure 2D; P<0.01), with the highest volume being at the most anterior region of the animal in front of the eyes (P<0.001) (Figure 2E5, E6, E11, E12, Figure S6). By using a panel of markers of differentiated cells [22], [24]–[28] we observed anterior blastemas with differentiation defects, especially the region that should contain the dorsal-most part of the brain ganglia and the eyes (Figure 2A, 2E13–E18, 2F and Figure S8A). This correlated with the accumulation of neoblasts and progeny in the same areas. Similarly in the regeneration of the digestive system, the anterior branch did not reach the level of the eyes and the posterior branches did not reach the most posterior part, being anastomosed instead of separated (Figure 2E15 and 2E18, Figure S8B). We observed that the most anterior tip of the animal displayed an ongoing lack of terminal differentiation (Figure 2E15, E18, 2F) having been transformed into a region of continuous growth and neoblast activity normally associated with deeper mesenchymal tissue regions or post-blastema regions after injury. Together our data suggests that loss of *Smed-smg-1* results in neoblast hyper-proliferation that extends to tissues normally devoid of cycling neoblasts. This in turns causes abnormal growth due to an uncontrolled accumulation of neoblasts and progeny, which prevent correct patterning and differentiation in these areas. Eventually, the over proliferation leads to hyperplasia, abnormal outgrowths and consequently death (Figure 2G).

### *Smed-smg-1(RNAi)* leads to lethal outgrowths which display several hallmarks of human cancers

**Figure 3:**
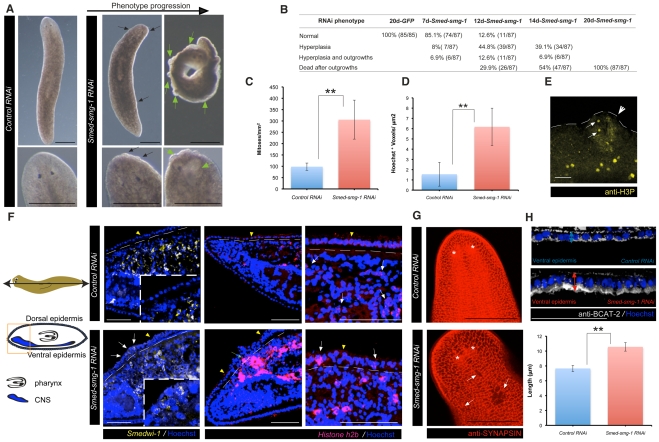
*Smed-smg-1(RNAi)* leads to lethal outgrowths during planarian homeostasis. A, B. Panels and table show *Smed-smg-1(RNAi)* phenotype progression compared to controls. Black arrows show epidermal hyperplasia and green arrows show outgrowths. B. Progression of the RNAi phenotypic characteristics at various time points (days) after the last injection. C. Mitotic numbers at 9 days after the last injection (n≥6 per condition). D. Volume of nuclei in 10 µm paraffin sections. Equivalent regions for at least 6 different planarians per condition were measured. E. Single confocal section of H3P^+^ cells (arrows) observed in an outgrowth (arrow head) for a *Smed-smg-1(RNAi)* animal at 9 days after the last injection (n = 7/8 displaying outgrowths had mitotic cells visible; see Video S3 for a whole worm view). F. The cartoons show how the sagittal paraffin sections were performed. Panels display the region of the orange square in the cartoons. Panels are maximum confocal projections of slices performed on 10 µm paraffin sections showing a monostratified dorsal and ventral epidermis in control animals and a multistratified dorsal epidermis in the case of *Smed-smg-1 (RNAi)* animals (yellow arrowheads). *Smed-smg-1(RNAi)* animals show *Smedwi-1* ^+^ cells in outgrowths (white arrows; n = 2/4 planarians), *h2b* ^+^ cells infiltrating to the epidermis (green arrow) and disrupting the integrity of the basement membrane (dotted white line) and at the multilayered epidermis (white arrows; n = 3/4 planarians). G. Dorsal-most confocal sections of the planarian head showing the subepidermal nerve plexus stained by synapsin where several disruptions can be observed in *Smed-smg-1(RNAi)* animals (arrows). Asterisks indicate the position of the eyes. H. The panels and graph show that *Smed-smg-1(RNAi)* animals have bigger ventral epidermal cells (hypertrophy) than controls. Panels display the ventral epidermis (deconvolved confocal maximum projections of slices performed on 10 µm paraffin sections). Double arrows show the distance from the basement membrane until the outer membrane of the epidermal cells (stained with anti-BCAT-2). See Material and methods for details about quantification method. Error bars for all the graphs are s.d from the mean and asterisks indicate P<0.01 using two-tailed Student's test with equal sample variance. Black scale bars indicate 300 µm and white scale bars indicate 50 µm.

We wished to understand the cellular nature of events leading to ectopic outgrowths in more detail to try and shed more light on how *smg-1* might regulate these processes in normal tissue. In order to investigate if *Smed-smg-1* was also regulating normal homeostatic control of neoblast proliferation we observed *Smed-smg-1(RNAi*) animals without amputation. These animals displayed neoblast hyper-proliferation (P<0.01) and general hyperplasia (P<0.01) accompanied by a profound anatomical disruption (Figure 3A, 3B, 3C, 3D and Figure S9A). Control planarians had typically well-defined monostratified epithelial cells characterised by larger columnar dorsal epithelial cells and smaller cuboidal ventral epithelial cells. However, the dorsal epidermis of *Smed-smg-1(RNAi)* animals appeared multistratified with numerous outgrowths causing planarians (n = 87/87) to die at around 20 days after the last injection (Figure 3B). Significantly, we observed *Smedwi1* ^+^, *histone H2B* ^+^ and anti-H3P^+^ neoblasts abnormally located in outgrowths, infiltrating the epidermis, disrupting the subepidermal nerve plexus and the integrity of the basement membrane (Figure 3E, 3F, 3G and Video S3). However, we never observed *Smed-NB.21.11e* ^+^ (n = 0/6 planarians) and only rarely *Smed-AGAT-1* ^+^ cells (Figure S9B). As for regeneration we also observed proliferation in front of the eyes (Figure S9C). Additionally, ventral epithelial cells were over 20% longer along the dorsal ventral axis than in control animals, indicative of cellular hypertrophy in a post-mitotic cell population (Figure 3H).

As just a tiny wound is enough to start the first mitotic response [8] one possibility would be that the homeostasis phenotype observed is also a response to the injury caused by RNAi by injection. To test this we performed an RNAi experiment by feeding, which does not involve causing injury to the planarians (Figure S10A and S10B). We observed the same phenotypic consequences that we observed for RNAi by injection. We observed that the increased mitotic response usually induced by feeding planarians [29] was higher in *Smed-smg-1 RNAi* animals than in controls (P<0.01) (Figure S10C). These data indicate that the mitotic response induced by feeding is also regulated by *Smed-smg-1* suggesting that both injury and growth responses require normal SMG-1 levels to place a brake on proliferation. Currently the methodology of RNAi delivery makes it impossible to ascertain whether loss of SMG-1 causes increased proliferation without an initial stimulus, such as wounding, amputation or feeding.

Altogether these data indicate that *Smed-smg-1* is necessary for controlling the proliferation of neoblasts and spatially restricting the cycling neoblast compartment during regeneration and homeostasis.

### Planarian mTORC1 is required for the initial response to injury and blastema formation

**Figure 4:**
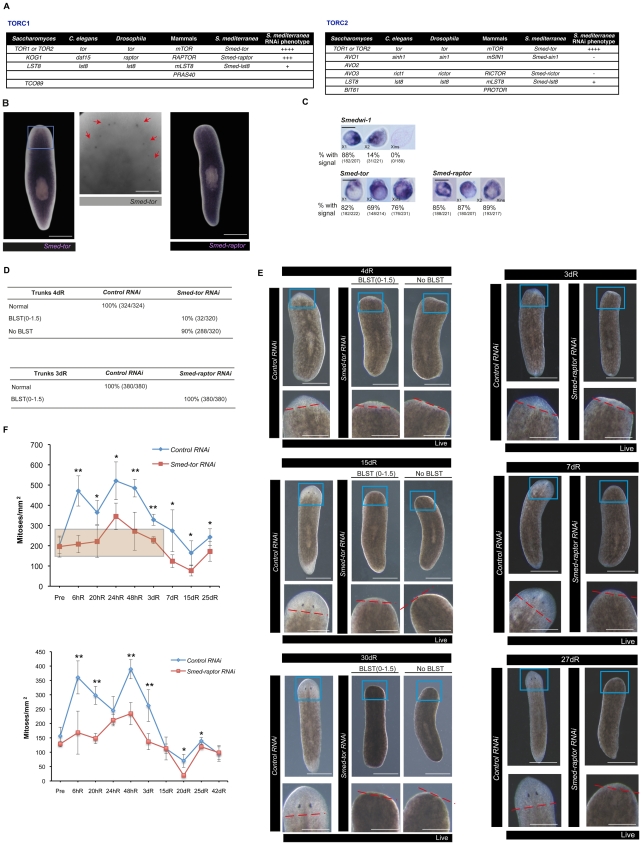
*Smed-tor* and *Smed-raptor* are necessary for the response to injury and blastema growth. A. Tables that summarise the components of mTORC1 and mTORC2 found in *S. mediterranea*. +++ indicates strong phenotype, ++ indicates weak phenotype, + indicates very weak phenotype, − indicates no phenotype after RNAi experiments. B. *Smed-tor* and *Smed-raptor* are broadly expressed in the planarian body. A high magnification of the head region (blue square) shows that *Smed-tor* is also expressed in a subset of neurons in the brain ganglia (red arrows) (n = 30/30 in three independent experiments). C. ISH on FACS-isolated cells. Representative results are shown. Percentages are signal-positive cells. *Smedwi-1* was used as a positive control and neoblast marker. *Smed-tor* and *Smed-raptor* are expressed in all three populations. D. Table that summarises the phenotypes observed in trunks at 4 dR for *Smed-tor* and 3 dR for *Smed-raptor*. BLST indicates blastema. Phenotype classification is according to previous published data [63]. E. Panels show representative planarians at different times of regeneration. *Smed-tor(RNAi)* planarians either show a small blastema (the biggest blastema observed is shown (BLST(0–1.5) or no blastema at all. *Smed-raptor(RNAi)* show smaller blastemas than controls (the biggest blastema observed is shown (BLST(0–1.5). The blastema does not grow further during regeneration, compared to controls. Planarians never showed signs of ventral curling or regression, typical features observed when abrogating genes expressed in neoblasts [22], [24] and were never depleted of neoblasts. The blue square represents the area of high magnification. The dotted red line defines the blastema. F. Mitotic numbers during different time points of regeneration and representative images showing H3P staining. Error bars are s.d from the mean and asterisks indicate P<0.05 (one asterisk) or P<0.01 (two asterisks) using two-tailed Student's test with equal sample variance (n≥5 planarians per time point in at least two independent experiments). Scale bars indicate 300 µm in B, 50 µm in B high magnification, 10 µm in the isolated cells in C, 300 µm in E and 150 µm in the E high magnifications.

mTOR signalling has been shown to control both growth and homeostasis in a number or organisms [30]–[32]. Previous RNAi experiments with homologs of PTEN in planarians, a known suppressor of mTOR signalling, have been shown to also lead to abnormal outgrowths thus implicating mTOR in controlling planarian growth [33]. We first characterised all the components of TORC1 and TORC2 in planarians (Figure 4A). We found the three main components of mTORC1, m*TOR*, the regulatory-associated protein of TOR called *RAPTOR* and LST8 in *S. mediterranea*, which we named *Smed-tor* and *Smed-raptor* and *Smed-lst8*, respectively (Figure 1, Figure 4, Figure S1, Figure S11 and Figure S12A; GenBank Accession Numbers JF894290, JF894291 and JN815261). We also identified the two main components of mTORC2, *RICTOR* and *SIN1*, which we named *Smed-rictor* and *Smed-sin1*, respectively (Figure S13A; GenBank Accession Numbers JN815259 and JN815260).

Consistent with their broad organismal role we observed that, in addition to being expressed broadly in the planarian body, *Smed-tor* and *Smed-raptor* were expressed in most neoblasts (Figure 4B and 4C). Abnormal neoblast proliferation in *Smed-tor* RNAi animals has been already described [8]. Although *Smed-tor(RNAi)* animals were able to close wounds after amputation, they were not able to form blastemas, even after more than 25 dR (Figure 4D and 4E). These animals lacked the first mitotic regeneration peak (P<0.01) and mitotic levels during regeneration were lower than controls (P<0.05) (Figure 4F). This difference was not due to a decrease in the number of neoblasts present before amputation (Figure S5), suggesting that the effects we observe are related to the control of mitotic responses to wounding and subsequent regeneration.

Consistent with RAPTOR phenocopying TOR in other organisms [34], *Smed-raptor(RNAi)* induced the same phenotype as *Smed-tor(RNAi)*, albeit with a weaker penetrance (Figure 4D). Although qPCR experiments showed that RNAi experiments downregulated *Smed-lst8* expression in a similar way to *Smed-raptor* after RNAi (Figure S12C), we observed a relatively weak phenotype that was nonetheless in agreement with the phenotypes described for *Smed-tor* and *Smed-raptor* (Figure S12B). RNAi for TORC2 components *Smed-rictor* and *Smed-sin1* did not show any apparent phenotype after more than 40 days of regeneration, even after three rounds of RNAi injections or combining RNAi of both genes (Figure S13B). The mRNA levels were however downregulated at similar values as in *Smed-raptor* RNAi experiments or *Smed-smg-1* RNAi experiments (Figure S13C).

**Figure 5:**
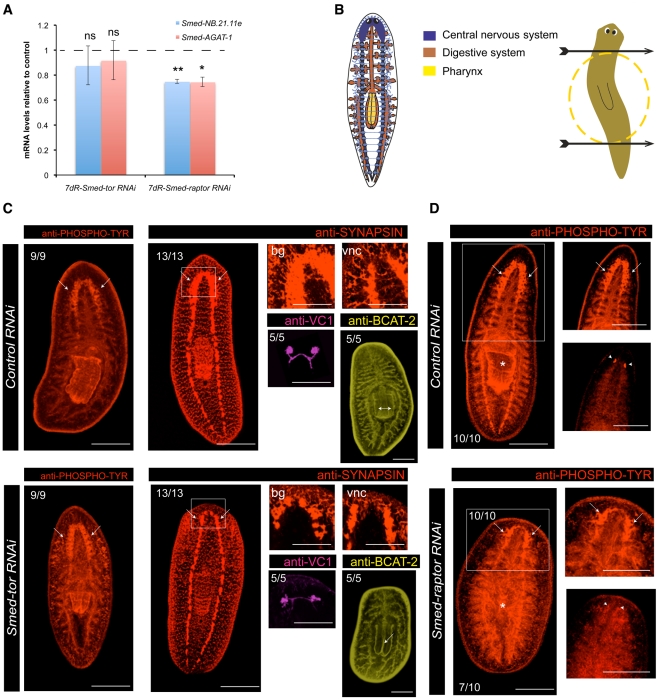
*Smed-tor* and *Smed-raptor* show similar levels of neoblast progeny as controls and are able to differentiate structures at anterior wounds. A. Relative expression of *cat-2* marker *Smed-NB.21.11e* and *cat-3* marker *Smed-AGAT-1* at 7 days of regeneration after either *Smed-tor* or *Smed-raptor* RNAi experiments. Expression levels are relative to *gfp* RNAi injected animals (dotted line). Error bars are s.d from the mean. Asterisks indicate P<0.05 (one asterisk), P<0.01 (two asterisks) and ns indicates “not significant” using two-tailed Student's test with equal sample variance and relative to expression in control animals. B. The cartoon on the left is a representation of the central nervous system (CNS in blue), the digestive system (brown) and the pharynx (yellow) in planarians. The cartoon on the right shows the levels of transverse amputation performed in the experiment (arrows) and highlights the trunk piece (dotted red circle), which was kept to follow anterior and posterior regeneration in all the experiments. C. The panels show staining for different markers of differentiation in the anterior wounds in control and *Smed*-tor RNAi animals. Anti-PHOSPHO-TYROSINE labels the CNS, digestive system, pharynx and eyes. The arrows highlight the presence of brain ganglia in both control and *Smed-tor* RNAi at anterior wounds in 12 dR trunks. Anti-SYNAPSIN labels the synapses at the CNS. The white square indicates that this region is magnified in the panels in the right and highlights the presence of brain ganglia in both control and *Smed-tor*, however underdeveloped in *Smed-tor* RNAi 7 dR trunks. bg, brain ganglia; vnc, ventral nerve cords. Anti-VC1 staining shows that controls and *Smed-tor* RNAi animals have differentiated eyes at the anterior wound site at 12 dR trunks. Anti-BCAT-2 staining shows that the lumen of the pharynx in *Smed-tor* RNAi animals is reduced (double arrow) compared to controls (arrow). D. Panels show 15 dR trunks. The panels show staining for anti-PHOSPHO-TYROSINE as a marker for CNS, digestive system and eyes in the anterior wounds of control and *Smed-raptor* RNAi animals. The white square indicates that this region is magnified in the panels in the right and highlights the presence of brain ganglia in both control and *Smed-raptor* RNAi animals (arrows), however underdeveloped in *Smed-raptor* RNAi animals (10/10). Arrowheads indicate the presence of eyes. The asterisk indicates the position of the pharynx, which is disorganized in *Smed-raptor* RNAi animals compared to controls (7/10). Scale bars indicate 300 µm.

Interestingly, it has already been shown that mTOR signalling is important for the proper balance of stem cell self-renewal and differentiation. For instance, mTOR downregulation has been shown to suppress embryonic stem cell self-renewal while enhancing endodermal and mesodermal differentiation [35]. We wanted to investigate if down-regulation of *Smed-tor* and *Smed-raptor*, in addition to decreasing, proliferation would enhance differentiation. We first monitored the production of differentiating neoblast progeny and found them to be as controls for *Smed-tor RNAi* animals (P>0.05) and reduced to around 75% for *Smed-raptor RNAi* (Figure 5A), the difference being likely due to the weaker phenotype shown by *Smed-raptor* RNAi.

We next assessed if stem cell differentiation was proceeding at the wound site. Although *Smed-tor* and *Smed-raptor* RNAi planarians showed defects in pharynx maintenance during regeneration at 7 dR (Figure 5B–5D, Video S4 and Video S5), they were able to restore missing structures at the wound site, but this occurred entirely within old tissues and without making a regenerative blastema (Figure 5C and 5D). Observation of *Smed-tor* and *Smed-raptor* RNAi planarians demonstrated they moved in a manner consistent with having a clear anterior to posterior polarity, consistent with correct differentiation at wound sites (Video S4 and S5). These results are in agreement with the observation of progeny production and show that neoblasts can differentiate to replace missing structures in spite of the absence of a blastema (Figure 5C and 5D).

Together these results show that mTORC1 regulates the initial mitotic response to injury or amputation and that loss of mTORC1 prevents correct blastema formation. mTORC1 down-regulation does not prevent differentiation at the wound site and missing structures can be differentiated within existing tissue without the requirement for the formation of a regenerative blastema. Taken together these data indicate that mTORC1 is necessary for both proliferative response to injury and for blastema formation and growth. Mechanisms controlling differentiation and formation of missing tissues appear to be intact suggesting that neither mTORC1 nor regenerative blastemas are required for their replacement.

### *Smed-smg-1* and mTORC1 act antagonistically in planarians

Given the mirrored effects of *Smed-smg-1* RNAi and mTORC1 RNAi with respect to proliferation, blastema formation and growth, and differentiation we wished to assess if *Smed-smg-1* phenotype manifestation required mTORC1 components or vice versa.

**Figure 6:**
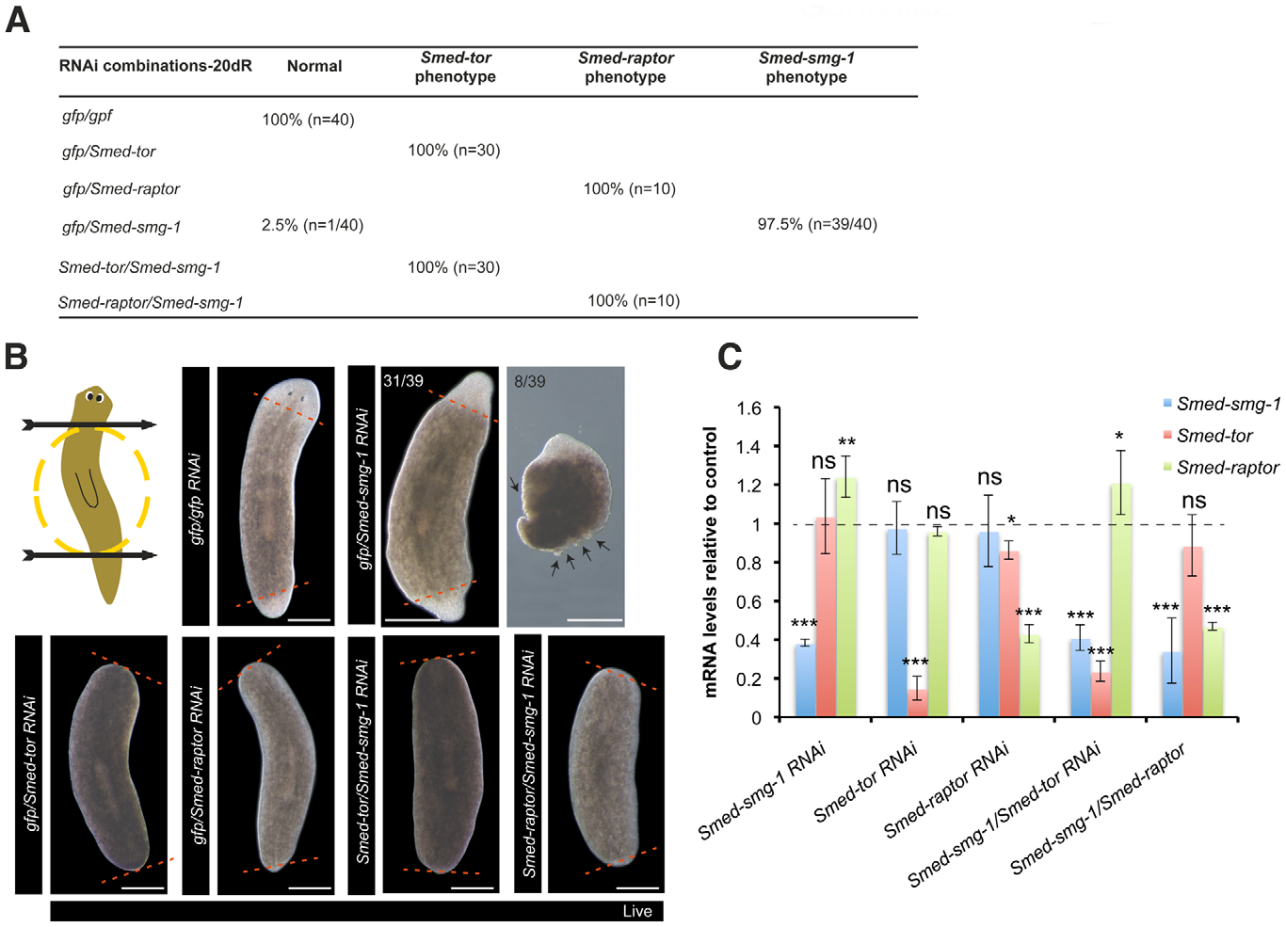
mTORC1 is necessary for *Smed-smg-1* RNAi phenotype. A. Table summarising the phenotypes observed after combinatorial RNAi experiments of *Smed-smg-1* with the members of TORC1. B. Panels show that double RNAi experiments of *Smed-smg-1* with either *Smed-tor* or *Smed-raptor* lead to lack of blastema formation, the same phenotype that is obtained after RNAi of either *Smed-tor* or *Smed-raptor* with *gfp*. Red dotted lines define the blastema. Arrows indicate outgrowths. C. Relative expression of *Smed-smg-1*, *Smed-tor* and *Smed-raptor* at 7 days of regeneration after single or double RNAi experiments. Expression levels are relative to *gfp* RNAi injected animals (dotted line). Similar levels of down regulation for the different genes are observed in single or double RNAi experiments (P>0.05). Error bars are s.d from the mean and asterisks indicate P<0.05 (one asterisk) or P<0.01 (two asterisks) or P<0.001 (three asterisks) using two-tailed Student's test with equal sample variance and relative to expression in control animals. Scale bars indicate 300 µm.

We performed double RNAi experiments with *Smed-smg-1* and mTORC1 components. Double RNAi experiments combining *Smed-smg-1(RNAi)* with either *Smed-tor(RNAi)*, *Smed-raptor(RNAi)* or *Smed-lst8(RNAi)* all showed the phenotype of *Smed-tor/Smed-raptor/Smed-lst8*. Double RNAi with *Smed-smg-1/gfp (RNAi)* only displayed the *Smed-smg-1(RNAi)* phenotype (Figure 6A, 6B and Figure S12B). We performed qPCR experiments to confirm that the mRNA levels of *Smed-smg-1* and planarian mTORC1 components were downregulated to similar values in both single and double RNAi experiments (P>0.05) (Figure 6C and Figure S12C). These results indicate that *Smed-tor*, *Smed-raptor* and *Smed-lst8* function are required for *Smed-smg-1(RNAi)* to drive uncontrolled growth.

**Figure 7:**
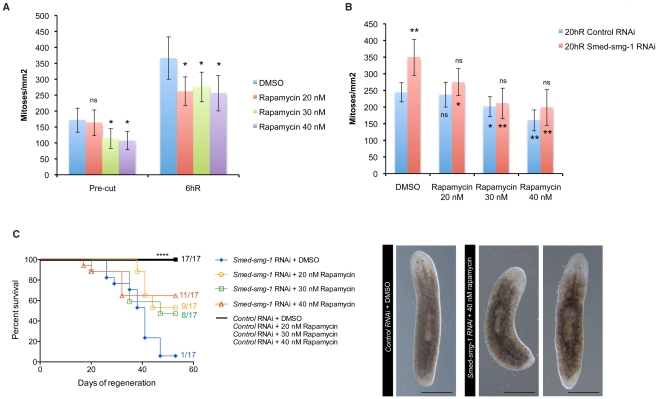
Rapamycin decreases neoblast proliferation and increases the survival rate in *Smed-smg-1* RNAi animals. A. Number of H3P positive cells per mm^2^ of un-cut planarians or 6 hR planarians that were previously treated with daily injections of DMSO, 20 nM rapamycin, 30 nM rapamycin or 40 nM rapamycin during 2 weeks. Error bars are s.d from the mean, the asterisk indicate P<0.05 while ns indicates “not significant” (P>0.05) using two-tailed Student's test with equal sample variance relative to the corresponding DMSO treated planarians. 6 hR shows an increased mitotic peak respect to the pre-cut condition despite the treatments (P<0.01). No significant differences (P>0.05) are observed either between the 30 nM and 40 nM rapamycin treatment in the pre-cut planarians or between the different rapamycin treatments in the 6 hR planarians. B. Number of H3P positive cells per mm^2^ in control RNAi and *Smed-smg-1* RNAi planarians at 20 hR that were treated with daily injections of DMSO, 20 nM rapamycin, 30 nM rapamycin or 40 nM rapamycin. Outside the bars two asterisks (p<0.01) show significant differences while ns (p>0.05) shows no significant differences relative to the control RNAi animals. The asterisks inside the blue bars show significance relative to the 20 hR control RNAi worms DMSO treated whereas inside the red bars the significance is shown relative to the 20 hR *Smed-smg-1* RNAi worms DMSO treated. Error bars are s.d from the mean and asterisks indicate P<0.05 (one asterisk) or P<0.01 (two asterisks) or P>0.05 (ns) using two-tailed Student's test with equal sample variance. ≥8 planarians per time point. C. Kaplan-Meier curves demonstrate increased survival of *Smed-smg-1* RNAi animals when treated with 20 nM, 30 nM or 40 nM rapamycin. Four asterisks indicate p<0.0001 with log-rank analysis of survival curves. Representative images of surviving animals after *Smed-smg-1* RNAi and treated with 40 nM rapamycin are shown at 45 dR. Scale bars indicate 300 µm.

mTOR signalling has been shown to be a critical pathway involved in tumour growth being the main target in the development of anti-cancer therapies. The macrolide rapamycin is a natural compound that was discovered as the first inhibitor of mTORC1. Rapamycin has shown promising results against some cancers like renal cell carcinoma or where PTEN is deleted, like endometrial cancers [36], [37]. Interestingly, it has also been shown to prevent the abnormalities produced by *Smed-PTEN* loss of function in planarians [33]. Therefore, rapamycin treatment experiments represent an extra tool for showing that the phenotype resulting from loss of *Smed-smg-1* results from overactive mTOR signalling. We first injected planarians with daily doses of rapamycin (20 nM, 30 nM or 40 nM) to check if rapamycin alone can affect proliferation during homeostasis and regeneration and mimic the effect of RNAi of planarian mTORC1 components. We quantified H3P^+^ cells 2 weeks after the daily treatment. Although we could not observe any morphological abnormalities, planarians displayed a significant decrease (P<0.05) in basal proliferation when treated with 30 nM or 40 nM rapamycin (Figure 7A). Although planarians formed normal regenerative blastemas and did not display any external phenotypes we found that the low dose of 20 nM of rapamycin was sufficient to decrease the mitotic levels with respect to DMSO treated controls at 6 h of regeneration. Rapamycin treated animals did however still display a significant mitotic response to amputation compared to the pre-amputation levels of proliferation (Figure 7A, P<0.01). Our data indicates that rapamycin treatment alone affects basal neoblast proliferation but does not specifically eliminate the mitotic response to injury or the normal blastema formation displayed in rapamycin treated planarians.

We next combined daily doses of rapamycin with RNAi experiments. Planarians were injected with rapamycin two days previous to the RNAi injections (*gfp* or *Smed-smg-1*), with amputation at day 8 after the first RNAi injection and continuing rapamycin injections stopped on day 25 when outgrowths were obvious in control DMSO+*Smed-smg-1* RNAi injected planarians. Some planarians were fixed at 20 h after amputation to observe proliferation at a time when the levels of proliferation are at the maximum in *Smed-smg-1* RNAi animals (Figure 2C). We observed that 20 nM rapamycin was able to decrease proliferation of *Smed-smg-1* RNAi animals to normal values (P>0.05) (Figure 7B). *Smed-smg-1* RNAi planarians treated with rapamycin showed the same unpigmented blastema as DMSO treated animals. However, 50% of the planarians treated with 20 or 30 nM rapamycin and more than 60% of the planarians treated with 40 nM rapamycin showed some differentiation in the blastemas and survived as long as the DMSO+*gfp* treated animals (Figure 7C). Our data indicates that rapamycin is able to reverse the effects of *Smed-smg-1(RNAi)* by decreasing proliferation and preventing the formation of outgrowths in about 50% of *Smed-smg-1(RNAi)* animals.

## Discussion

mTOR signalling has central control of cell growth and proliferation in all eukaryotes analyzed so far [31]. Consistent with mTOR signalling regulating growth, human genetic defects that are associated with upregulated mTOR activity manifest as abnormal cell growth and proliferation. Indeed, many negative regulators of mTOR signalling are known human tumour suppressors. As a result mTOR signalling is currently the most targeted signalling pathway in drug development for the treatment of cancers.

It is known that mTOR signalling is essential for the early developmental programmes of metazoans. For instance *Drosophila* or *C. elegans* TOR loss-of-function mutations leads to developmental arrest [38], [39] and homozygous mTOR^−/−^ mouse embryos die shortly after implantation due to impaired cell proliferation in both embryonic and extra-embryonic compartments [40]–[42]. mTOR itself forms the core of two different complexes and of these TOR complex 1 is thought to be responsible for regulating cell growth and proliferation [31]. We show that planarian *Smed-tor*, *Smed-raptor* and *Smed-lst8* homologs of *TOR*, *RAPTOR* and *LST8* respectively and members of TORC1, are necessary for blastema growth during regeneration (Figure 4 and Figure S12). RNAi experiments for *Smed-tor* and *Smed-raptor* result in a lack of the first mitotic peak and blastema during regeneration and show lower general levels of proliferation through the whole regenerative process. Recently it has been shown that planarian amputation triggers two peaks in neoblast mitoses early in regeneration. The first mitotic peak is a body-wide response to any injury and the second response is induced only when injury results in missing tissue [8]. RNAi experiments for *Smed-tor* or *Smed-raptor* show that these animals lack the first mitotic peak of regeneration and thus lack a response to injury. Our results suggest both that the first mitotic peak is necessary for blastema formation and that this first mitotic peak requires the activation of mTOR signalling. The second mitotic peak is a response to missing tissue and it triggers neoblast differentiation [8]. Although reduced, *Smed-tor* and *Smed-raptor* RNAi planarians did show a second mitotic peak (Figure 4F) and some ability to restore missing structures at the wound site within old tissues without making a blastema (Figure 5C and 5D). The role of mTOR signalling in regeneration [43] and in the regulation of stem cells [44], [45] is just starting to be elucidated. Our results support a key role for mTOR signalling in controlling stem cell proliferation and growth during regeneration.

In this study we have also characterised the planarian homolog of *hSMG-1*. SMG-1 has widely been linked to NMD [15] and SMG-1 has been also shown to have functions independent of NMD, mostly related to cellular stress responses [46]. However, its physiological roles are not yet well understood and only few studies have been performed *in vivo*. In *C. elegans* and *Drosophila* Smg-1 is dispensable for survival and development while other components of NMD in these organisms are essential [15], [47]. In mice, SMG-1 is dispensable for implantation and gastrulation but is critical thereafter for basic differentiation, a phenotype presumed to reflect its function in mediating NMD [48].

Here we have showed that *Smed-smg-1(RNAi)* results in higher and extended mitotic responses to injury at 6 hR. Following this levels of proliferation remain raised and this contributes to these animals undergoing continuous growth within the blastema. Cells fail to terminally differentiate, proliferating cells remain in aberrantly high numbers and accumulate inappropriately within the blastema. Thus, *Smed-smg-1* in contrast to planarian mTORC1 components is required for restricting the response to amputation and the growth of the blastema. Our data suggest that uncontrolled growth occurs in a gradient along the dorso-ventral axis, increasing from ventral to dorsal regions. Several indications support this observation. In anterior regenerating blastemas, cycling neoblasts accumulate initially in the ventral part of the animal, and this is the only area in which some correct terminal differentiation is observed. In more dorsal blastema regions less differentiation is observed. Instead, we observe the presence of cycling neoblasts invading the dorsal-most part of the brain, a lack of terminally differentiated cells and accumulation of neoblast progeny. This continuous growth of the blastema eventually leads to abnormal and lethal outgrowths, with all the outgrowths initially formed at the dorsal level consisting of cycling neoblasts and progeny. These observations suggest that normal planarian growth may follow a ventral to dorsal pattern. During homeostasis, we observed uncontrolled proliferation that also results in lethal ectopic outgrowths. To investigate the possibility that the phenotype observed during homeostasis is also a response to the injury caused after RNAi injections we performed experiments of RNAi by feeding. However, we also observed that *Smed-smg-1* regulates the mitotic response to feeding. Thus we were unable to ascertain whether or not *Smed-smg-1* also has a role as a homeostatic brake for growth control or whether it is specific to providing a brake to stimuli that promote growth. Nevertheless, we have uncovered a new role for *Smg-1* in stem cell regulation, regeneration and growth. It will be very interesting to investigate the possible evolutionary conservation of these novel roles in other systems.

It has already been shown that planarians provide a model system with which to study tumour suppression and adult stem cell lineages *in vivo*. RNAi experiments on the planarian homologs of several known human suppressors such as PTEN or p53 have also shown to lead to outgrowths [33], [49]. Although the *Smed-smg-1(RNAi)* phenotype shares some characteristics with *Smed-PTEN* or *Smed-p53* such as hyper-proliferation, hyperplasia and breakdown of the sub-epithelial basement membrane that surrounds the animal, it also has characteristics not previously described for other planarian RNAi phenotypes. Firstly, we observe the presence of cycling neoblasts in front of the eyes, a region devoid of neoblasts. Secondly we observe hypertrophy of the ventral epidermis.

We describe almost polar opposite roles for planarian mTORC1 and *Smed-smg-1* in planarian regeneration. Firstly, mTORC1 and *Smed-smg-1* have opposing effects on proliferation and blastema growth (Figure 2, Figure 4). Secondly, we found that while *Smed-tor* RNAi maintains differentiation at the wound site in the absence of blastema formation, terminal differentiation is impeded in *Smed-smg-1* RNAi animals (Figure 2, Figure 5). Combinatorial RNAi experiments showed the *Smed-smg-1(RNAi)* uncontrolled growth requires mTOR signalling to manifest (Figure 6). Supporting these results, experiments combining rapamycin and RNAi also showed that rapamycin is able to prevent the outgrowths and increase the survival rate of *Smed-smg-1* RNAi planarians in about 50% of animals. While these experiments confirm that loss of mTORC1 generally inhibits growth it also raises the possibility that *Smed-smg-1* has either a direct or indirect interaction with mTOR signalling. The broad expression patterns of *Smed-smg-1*, *Smed-tor* and *Smed-raptor* in the planarian body, with similar distributions of expression in neoblasts, progeny and differentiated cells also suggests this is a possibility. Future genetic and biochemical work in cell lines and other models systems will help elucidate the relationship between SMG-1 and mTORC1 signalling.

Previously published data showed that rapamycin does not affect basal proliferation in planarians [33]. However, only homeostatic planarians were tested injecting doses of 20 nM rapamycin. We also found that 20 nM does not affect proliferation during homeostasis. However, already 20 nM rapamycin was able to significantly decrease proliferation in regenerating planarians while 30 and 40 nM rapamycin doses reduced proliferation in both homeostatic and regenerating planarians. We observed differences between the rapamycin treatment and the inhibition of mTORC1 in planarians. Rapamycin was able to decrease the general levels of planarian proliferation without abolishing the mitotic response to injury and did not affect blastema formation and growth. Significantly, rapamycin does not completely inhibit mTORC1 activity in mammalian cells and clearly affects some TORC1 targets more than others, depending on cell type and context [50]–[53].

Altogether our results indicate a novel function for SMG-1. Our findings support a model where SMG-1 acts as break on growth and proliferation. The altered patterns of proliferation and the presence of abnormal invasive cells disrupting the epithelial-mesenchymal interactions we have observed in *Smed-smg-1(RNAi)* planarians are hallmarks in the early progression of most human cancers. To date reports from the COSMIC database describe mutations of SMG-1 in human breast cancer cell lines and hSMG-1 RNA is detected only at low levels in lung carcinoma and melanoma cell lines [14], [20]. These reports together with our new functional findings indicate SMG-1 is likely to be a potential human tumour suppressor gene product. Given this new physiological role for *Smg-1* it will now be important to investigate whether mutations in hSMG-1 results in cell growth and/or invasiveness contributing to the aetiology of cancers.

The defects observed after *Smed-smg-1(RNAi)* may not result from defects in the previously characterised role in the NMD pathway. Very recently, a planarian RNAi screen has shown that several components of the NMD pathway in planarians either show no phenotype after RNAi or show a different one to that observed in *Smed-smg-1(RNAi)* [54]. This suggests that the phenotypes observed for *Smed-smg-1* may be independent of NMD, alternatively our results may reflect unknown roles for a subset of NMD activities requiring SMG-1 but not other NMD components. A precise understanding of the mechanism(s) by which SMG-1 affects impacts growth and proliferation in physiological conditions may be important in the development of novel cancer therapies and other diseases. The identification of a candidate tumour suppressive function for SMG-1 further validates the planarian system as a source of novel insights relevant to human disease processes.

## Materials and Methods

### Animals

Planarians used in this work belong to the species *Schmidtea mediterranea* asexual strain and were maintained at 19°C in tap water treated with activated charcoal and buffered with 0.5 ml/L 1 M NaHCO3. Planarians were fed organic veal liver and starved for at least one week prior to experiments.

### Planarian gene identification and phylogenetic analysis

Protein sequences from different organisms (from yeast to human) were used to find planarian homologs from the *S. mediterranea* genome database and transcriptome [55], [56] via tBLASTn searches. Planarian homologs were then used for reciprocal BLAST to verify homology. We found three contigs encoding *Smed-smg-1* (Contig 8142, Contig 8791, Contig 7718), one contig encoding *Smed-tor* (Contig 497), two contigs encoding *Smed-raptor* (Contig 424 and Contig 569) one contig encoding *Smed-lst8* (Contig 55), 4 contigs encoding *Smed-rictor* (Contig 2311, Contig 44763, Contig 91234 and Contig 10206) and 4 contigs encoding *Smed-sin1* (Contig 41273, Contig 15360, Contig 23280 and Contig 15360). Protein domains were predicted using InterProScan (EMBL-EBI) and PFAM (Wellcome Trust Sanger Institute). GenBank accession numbers for the genes reported here are: JF894292 *Smed-smg-1*, JF894291 *Smed-tor*, JF894290 *Smed-raptor*, JN815261 *Smed-lst8*, JN815259 *Smed-rictor* and JN815260 *Smed-sin1*. Using as queries previously described SMG-1 and TOR proteins from various metazoans, we performed tBLASTN and BLASTP searches against genomes and EST datasets of different organisms. Each gene was aligned independently using MAFFT v.6.850 [57], with the EINSI strategy. The resulting alignments were checked with Bioedit v. 7.0.9.0 [58] and edited with Geneious (available from http://www.geneious.com). For the full-length gene alignments of RAPTOR and the PIKK family, regions of ambiguous alignment were removed by Gblocks v.0.91b [59] using the “relaxed” options from the server version. Bayesian inference trees were inferred with a parallelized version of MrBayes v.3.1.2 [60], running 1,000,000 generations in 2 independent analyses with a sample frequency of 1 in 1000; the evolutionary model used in MrBayes was WAG+Γ+I (4 gamma categories+1 invariable). To obtain the consensus tree and bayesian inference supports, 10% of the generations were removed to discard trees sampled before likelihood values had reached a plateau. Maximum likelihood trees were inferred with RAxML v.7.2.6 [61], ran using the model LG+Γ+I (4 gamma categories+1 invariable); a random topology was used as starting tree and 1,000 bootstrap replicates were obtained.

### Planarian RNAi experiments

We generated 400 bp–700 bp templates with T7 promoters appended to both strands from *Smed-smg-1*, *Smed-tor*, *Smed-raptor*, *Smed-lst8*, *Smed-rictor* and *Smed-sin1*. We confirmed the single gene specificity of the chosen templates by BLAST search against the planarian genome. The primers used were as follows:

Smg-1-F: 5′-TGGCTGGAATTTGTTACGCATCACT-3′

Smg-1-R: 5′-GTCGCATTTTTGGTTCGTTCAAGAA-3′

TorF: 5′-TAGTGTCAATCTGCAAAAGGCCTGG-3′

TorR: 5′-GAACGACTCCACTAGCGGCTTCTTC-3′

RaptorF: 5′-GAATTCTTCATCAATGGACAAC-3′

RaptorR: 5′-TGTGATATATTTCAGGGAGCTG-3′

Lst8F: 5′- TATGACATTTTATTTGCCACTGGTG-3′

Lst8R: 5′- GCCTTCCACATTCATAGAAAGA-3′

RictorF: 5′- AGGCGTTATTCAACAAAATTTC-3′

RictorR: 5′- TATCGAACCATAAGGATGACAA-3′

Sin1F: 5′- GTTTTGGAAACGAAAGTGTCAG-3′

Sin1R: 5′- TATCGAGTTCCTTGTTTGGTTC-3′

Double-stranded RNA (dsRNA) was synthesized by *in vitro* transcription (Roche) and injected into planarians as described previously [62]. All animals were injected ventrally at different pre- and post-pharyngeal positions to rule out the possibility that the defects observed were related to the place of injection. Control animals were always injected with *GFP* dsRNA, a sequence not present in the planarian genome. Different schedules of RNAi injections were tried until a phenotype was observed. For *Smed-smg-1* RNAi regeneration experiments planarians were injected following the standard schedule of three consecutive days of injections and amputated five days after the last injection (one round of injections) [62]. For *Smed-smg-1* RNAi homeostasis experiments, planarians were injected following the standard schedule of three consecutive days of injections and two weeks after the first injection were injected again for other three consecutive days. *Smed-tor*, *Smed-raptor* and *Smed-lst8* required two rounds of injections with a gap of 10–14 days of regeneration after the first amputation and before starting the second round of injections. For *Smed-rictor* and *Smed-sin1*, three rounds of injections with a gap of 14 days of regeneration after every amputation did not give any phenotype. Because of the requirement of two rounds of injections for *Smed-tor*, *Smed-raptor* and *Smed-lst8* double injections of *Smed-tor/Smed-smg-1(RNAi)*, *Smed-raptor/Smed-smg-1(RNAi)* and *Smed-lst8/Smed-smg-1(RNAi)* were performed single for *Smed-tor/Smed-raptor/Smed-lst8* during the first round and double with *Smed-smg-1* during the second round. Planarians were always amputated at the pre and post-pharynx level to observe regeneration. For simultaneous silencing of two genes, dsRNAs were proportionally diluted and animals with the same dose of each single dsRNA were injected in parallel as controls, for instance for the *GFP/Smed-smg-1(RNAi)*, 1/2 *GFP* dsRNA+1/2 *Smed-smg-1* dsRNA was injected whereas a double dose of GFP was used for the controls (*GFP*/*GFP*(RNAi). To rule out that the phenotype observed was in part due to the starvation process of the worms during the long duration of the RNAi schedules, all the experiments involving *Smed-tor* or *Smed-raptor* were also performed feeding the worms 6–7 days after every amputation and showed the same RNAi phenotypes as during starvation.

dsRNAs for feeding were generated and fed to planarians as described previously [63]. Control animals were fed bacteria containing *gfp* cloned into the pPR242 vector [63]. The same region of the genes used for the injection experiments was used for the feeding experiments. Planarians were fed every 3 days a total of four times. In the regenerating experiments planarians were amputated 2 days after the last feeding in the same way as in the injection experiments. Only two feedings were delivered to the planarians to be analysed for the proliferation response to feeding.

### Real-time PCR

RNA was extracted using Trizol reagent (Invitrogen). cDNA was obtained from 2 µg of total RNA by using MMLV Reverse Transcriptase (Promega). Elongation Factor 2 (EF2) was used as an internal control. Two qPCRs per sample were performed. Each qPCR was performed with two biological replicates. Five animals were used per replicate, and each sample was replicated three times in each real-time PCR experiment. PCR reactions were performed using the SensiMix SYBR No-ROX Kit (Bioline). Reactions were aliquoted using a Corbett Robot (Corbett Robotics) and analysed with a Rotor Gene 6000 (Corbett, Qiagen). qPCR oligos for *Smed-NB.21.11e* and *Smed-AGAT-1* were used as already described [23]. The following gene specific oligos were designed from non-overlapping regions of the gene that were non-overlapping with sequence used for dsRNA:

Smg-1-QF: 5′-GATTGGACGATTAGAAATGGCT-3′

Smg-1-QR: 5′-ATAAACGAACTATGGCTTGTGG-3′

TorQF: 5′-ATCAACTCTATCAAGAACTGCC-3′

TorQR: 5′-GACCATCATCACCGATAATTGT-3′

RaptorQF: 5′-AAATGCTGAGGAAGATAGAGGT-3′

RaptorQR: 5′-ACGCAAATAGTTACGCCAAA-3′

Lst8QF: 5′- GATAGCACCACTGTTGTCAC-3′

Lst8QR: 5′- CAATCCCAAACCCATTTCATAC-3′

RictorQF: 5′- GTATCATGTCACTCATTTCCGT-3′

RictorQR: 5′- GCTTAAACCATTCCGTTGTTC-3′

Sin1QF: 5′- CACTGCGATATACAAAGGGTAA-3′

Sin1QR: 5′- AGATCGGTTTCTCTGCTCTT-3′

### Planarian *in situ* hybridization (ISH)

Digoxigenin-labelled RNA probes and fluorescein-labelled RNA probes were prepared by using an *in vitro* labelling kit (Roche, Basel, Switzerland). Single and double whole mount ISH and on paraffin sections were carried out as described previously [64]–[68]. Planarian ISH on dissociated cells were performed as previously described [69] and developed with NBT/BCIP.

### Planarian immunohistochemistry

Whole-mount immunohistochemistry was carried out as described elsewhere [70]. The following antibodies were used: anti-VC-1 [28], specific for planarian photosensitive cells (diluted 1/15,000); anti-SYNORF1 or anti-SYNAPSIN used as a pan-neural marker [26] (diluted 1/25, Developmental Studies Hybridoma Bank); anti-BCAT-2 [25], which labels the membrane of epithelial cells (diluted 1/2000); anti-TMUS13 [27], specific for myosin heavy chain (diluted 1/20); anti-Histone H3 phosphorylated at serine 10 [21] to detect mitotic neoblasts (diluted 1/500, Millipore) and anti-phospho-tyrosine [70] a neural marker that also labels the digestive system (diluted 1/200, Cell Signalling Technology, NEB). Alexa488-conjugated goat anti-mouse and Alexa568-conjugated goat anti-rabbit were used as secondary antibodies (Molecular Probes; diluted 1/400 and 1/100 respectively). Cavity slides were used to preserve the morphology of the outgrowths.

### Planarian cell sorting

Planarian cells from X1, X2 and Xins populations were isolated by fluorescence-activated cell sorting, using a Beckman-Coulter Ultra flow as previously described [71].

### Rapamycin treatment

Rapamycin treatment was performed as previously described [33].

### Imaging and quantification

Bright field and live images were observed through a Zeiss Discovery V8 (Carl Zeiss) or a Leica MZ16F (Leica) stereomicroscope and a Nikon Eclipse 80i microscope and recorded on a AxioCam MRC (Carl Zeiss), a Leica DFC 300Fx camera (Leica) or a Hamamatsu Orca-ER. Confocal laser scanning microscopy was performed for fluorescent samples with a Leica SP2 confocal microscope (CLSM) (Leica). Images were processed using ImageJ 1.43l or Adobe Photoshop CS5 software and compositions done on Adobe Illustrator CS5. *Smedwi-1* ^+^, *Smed-NB.21.11e* ^+^ or *Smed-AGAT-1* ^+^ voxels or *Smedwi-1* ^+^ cells were quantified by Volocity (Perkin Elmer) using confocal stacks deconvolved by the same program. *Smedwi-1* ^+^ cells were quantified in equivalent confocal stacks of 0.035 *mm^2^* in two different regions of the planarian: just in front of the pharynx and immediately posterior to the pharynx. Position of the eyes in Figure 2E was determined either by a white field image, the background of Hoechst in the eyes, for being negative when staining with *cat-2* and *cat-3* markers or for being positive to the marker anti-VC1 [28]. Z-projections and 3D reconstructive videos were also performed in Volocity after deconvolution. H3P quantifications were done on whole planarian confocal stacks and using the Object Counter 3D pluggin from ImageJ 1.43l. Hoechst^+^ voxels were quantified using the Voxel Counter pluggin from ImageJ. The length of ventral epidermal cells was calculated with ImageJ as the average of at least 8 different points of confocal stacks deconvolved using Volocity for at least 4 planarians. For all graphs, error bars represent standard deviation of the mean (sd) and asterisks indicate P<0.05 (one asterisk) or P<0.01 (two asterisks) using two-tailed Student's t-tests with equal sample variance.

### Accession numbers

All sequences associated with this study have been deposited in GenBank and have accession numbers JF894290 to JF894292 and JN815259 to JN815261.

## Supporting Information

Figure S1 SMED-SMG-1, the homolog of hSMG-1, and SMED-TOR, the homolog of mTOR, are members of the PIKK family of proteins. A. Schematic drawing of the domains present in all the members of the PIKK family of proteins. The proteins displayed are the ones present in humans. B. Maximum Likelihood phylogenetic tree of the PIKK+FATC domain in PIKK proteins. Phylogeny inferred with RAxML (GTR+Γ+I), numbers correspond respectively to Bootstrap supports and Bayesian inference Posterior Probabilities. A black dot indicates a clade with Bootstrap support superior to 95% and Bayesian Posterior Probabilities (PP) values of 1,0. Values under 50% or 0.5 are not indicated. The scale bar indicates the number of changes per site. For accession numbers corresponding to each terminal see Table S1.
(PDF)

Figure S2 *Smed-smg-1* is a bona fide *SMG-1*. A. Schematic illustration of the domains present on SMED-SMG-1 compared to hSMG-1. B. Multiple alignment (Blosum 62) for the FRB, PIKK and FATC domains of SMG-1 from several organisms. The critical tryptophan residue required for mTOR kinase activity [14] is conserved in SMG-1 indicated with an asterisk in the FRB alignment. hSMG-1 contains several highly conserved motifs found in all PIK-related kinases [13], [14]. A conserved ATP-binding site indicated by the asterisk in the PIKK domain is conserved in all the SMG-1 proteins from the different organisms displayed and motif I and motif II sites within the catalytic domain indicated by the blue lines are also conserved in all the SMG-1 proteins. Residues, whose substitution decreases hSMG-1 kinase activity, are labelled with an asterisk in the FATC alignment and are conserved in all the organisms displayed.
(PDF)

Figure S3 *Smed-smg-1* is required for correct blastema growth and to restrict the *category 1* compartment during regeneration. A. The cartoon shows the levels of transverse amputation performed in the experiment (arrows) and highlights the trunk piece (dotted yellow circle), which was kept to follow anterior and posterior regeneration in all the experiments. B. Panels show 7 dR trunks. Dotted red lines define the blastemas and the blue square shows the area displayed in the fluorescent panels. Control animals show an anterior blastema with a couple of eyes, where the pigmentary cup can be seen in the live images (black arrows) and the photosentitive cells and optic chiasm in anti-VC1 staining (white arrows and green arrow, respectively). *Smed-smg-1(RNAi)* animals show varying sizes of blastemas with a low degree of eye differentiation displayed in the live images (black arrows). The anti-VC1 staining shows the presence of underdeveloped photosensitive cells (white arrows) and no optic chiasma. Panels show maximum projections of the dorsal-most confocal sections, the medial-most confocal sections and the ventral-most confocal sections of anterior regeneration in 7 dR trunks. Asterisks indicate the position of the eyes. Panels show the distribution of neoblasts (*cat-1*), neoblast early progeny (*cat-2*) and neoblast late progeny (*cat-3*) markers (n≥4). The white arrow indicates the presence of cat 1 and H3P^+^ cells in the *cat-2* and *cat-3* compartments in front of the eyes in *Smed-smg-1(RNAi)* animals. Lower panels show a very undeveloped brain as seen by synapsin staining (dorsal-most maximum confocal projection) similar to the phenotype at 20 dR (Figure 2 in main manuscript). Scale bars indicate 300 µm.
(PDF)

Figure S4 During regeneration, *Smed-smg-1(RNAi)* animals with abnormal blastemas progressed to form outgrowths and die. A. Panels show some examples of planarians at 30–35 dR presenting hyperplasia and outgrowths. Green arrows indicate epidermal hyperplasia and black arrows indicate outgrowths. Scale bars indicate 300 µm. B. The cartoon represents a sagittal paraffin section. The orange square shows the area of the section displayed in the fluorescent panels. Panels are confocal maximum projections of slices performed on 10 µm paraffin sections. White arrows indicate the multilayered epidermis in *Smed-smg-1(RNAi)* animals (n = 10/10 planarians). Scale bar indicates 50 µm.
(PDF)

Figure S5 *Smed-smg-1(RNAi)* and *Smed-tor(RNAi)* animals show similar number of *Smedwi-1* ^+^ cells before amputation to control RNAi animals. *Smedwi-1^+^* cells quantification in 0.035 mm^2^ equivalent regions of the animals, anterior and posterior to the pharynx, prior to amputation in *Smed-smg-1* and *Smed-tor* RNAi animals. Error bars are s.d from the mean. No significant differences P>0.05 (ns) were observed in relation to control RNAi animals using two-tailed Student's test with equal sample variance. ≥5 planarians per time point.
(PDF)

Figure S6 *Smed-smg-1* RNAi animals show anterior expansion of *cat-1* and *cat-3* compartments. Volume in µm3 of *cat-1* and *cat-2* compartments in *Smed-smg-1* RNAi compared to control RNAi animals quantified from the region of the eyes until the tip of the head. Error bars are s.d from the mean and three asterisks indicate P<0.001 using two-tailed Student's test with equal sample variance relative to control RNAi animals.
(PDF)

Figure S7 *Smed-smg-1(RNAi)* animals show a posterior-dorsal expansion of the neoblast compartment at posterior-facing blastemas and an accumulation of late neoblast progeny at the tip of the blastema. The yellow square on the cartoon shows the area displayed in the fluorescent panels. Panels show maximum projections of 20 d posterior regenerating trunks. Panels show the distribution of neoblasts (*cat-1*), H3P^+^ cells, and neoblast late progeny (*cat-3*) markers (n = 6/6). The blue box indicates the area of high magnification. Scale bars indicate 300 µm and 50 µm in the high magnifications.
(PDF)

Figure S8 *Smed-smg-1(RNAi)* animals display differentiation problems during regeneration. A. The yellow square on the cartoon shows the area displayed in the fluorescent panels. Panels show maximum projections of the dorsal-most and the ventral-most confocal sections for the most developed brain observed at 20 dR planarians (stained with anti-SYNAPSIN). Control planarians for these images are seen in Figure 2E in main manuscript. B. The yellow square on the cartoon shows the area displayed in the fluorescent panels. Panels show maximum projections of 20 days posterior regenerating trunks. Anti-BCAT-2 shows the epithelia of the gut. Arrows indicate that the end of the posterior gut branches display anastomoses in *Smed-smg-1(RNAi)* animals but are not fused in controls (n = 6/6 versus n = 0/6 in controls). Double arrows show that the posterior gut branches are thicker in *Smed-smg-1(RNAi)* than controls (n = 6/6). It was not possible to determine from this experiment if the higher thickness is due to an increase in cell number or an increase in cell size. Scale bars indicate 300 µm.
(PDF)

Figure S9 Homeostatic *Smed-smg-1(RNAi)* animals show hyper-proliferation, mitoses in front of the eyes with late neoblast progeny rarely observed in outgrowths. A. Representative images showing H3P staining at 9 days after the last injection. Scale bars indicate 300 µm. B. The cartoon shows a sagittal paraffin section. The orange square shows the region represented in the panels. The arrows indicate *Smed-AGAT-1* ^+^ cells (n = 1/4 planarians). C. The cartoon indicates the area showed in the panels. Panels are confocal projections of anti-phospho-tyrosine immunofluorescence, pseudocolored according to the depth of the focal plane: dorsal-most sections are shown in magenta; deeper sections in green. *Smed-smg-1(RNAi)* animals display mitosis (white arrows) in front of the eyes (red arrows) (n = 3/7). Scale bars indicate 150 µm. Scale bars indicate 50 µm.
(PDF)

Figure S10 *Smed-smg-1* restricts the mitotic response to feeding. A, B. Tables show *Smed-smg-1(RNAi)* feeding phenotype progression compared to controls. d are days after the last injection. A. Homeostasis phenotypes. B. Phenotypes in trunks at 25 d of regeneration. C. Number of H3P positive cells per mm^2^ in control and *Smed-smg-1* RNAi planarians 1 d after feeding the corresponding dsRNAs. Error bars are s.d from the mean, the asterisks indicate P<0.01 using two-tailed Student's test with equal sample variance relative to the controls. n≥5.
(PDF)

Figure S11 SMED-TOR and SMED-RAPTOR conserved domains. A. Schematic illustration of the domains present on SMED-TOR compared to human mTOR. B. Schematic illustration of the domains present on SMED-RAPTOR compared to human RAPTOR. RNC domain accounts for RAPTOR N-terminal conserved domain.
(PDF)

Figure S12 *Smed-lst8* is a bona fide *LST8*, is broadly expressed in the whole planarian body and RNAi experiments show a very weak phenotype however similar to the ones obtained for *Smed-tor* and *Smed-raptor*. A. *Smed-lst8* is broadly expressed in the planarian body (n = 30/30 in three independent experiments). B. The cartoon shows the levels of transverse amputation performed in the experiment (arrows) and highlights the trunk piece (dotted red circle), which was kept to follow anterior and posterior regeneration in all the experiments. Panels show 16 dR trunks. Dotted red lines define the blastemas. *Smed-lst8* RNAi planarians show a reduced blastema compared to controls. Double *Smed-smg-1/Smed-lst8* RNAi experiments show *Smed-lst8* phenotype. C. Relative expression of *Smed-smg-1* and *Smed-lst8* at 10 days of regeneration after single or double RNAi experiments. Expression levels are relative to *gfp* RNAi injected animals (dotted line). Similar levels of downregulation for the different genes are observed in single or double RNAi experiments (P>0.05). Error bars are s.d from the mean. Asterisks indicate P<0.001 (three asterisks) and ns indicates “not significant” using two-tailed Student's test with equal sample variance and relative to expression in control animals. Scale bars indicate 300 µm.
(PDF)

Figure S13 *Smed-rictor* and *Smed-sin1* are bona fide *RICTOR* and *SIN1*, respectively. RNAi experiments of both genes either alone or combined showed no phenotype. A. E-value of the genes respect to the human homolog. B. The cartoon shows the levels of transverse amputation performed in the experiment (arrows) and highlights the trunk piece (dotted red circle), which was kept to follow anterior and posterior regeneration in all the experiments. Panels show 20 dR trunks. Dotted red lines define the blastemas. *Smed-rictor*, *Smed-sin1* or *Smed-rictor/Smed-sin1* RNAi did not show any phenotype even after three rounds of RNAi and regeneration. C. Relative expression of *Smed-rictor* and *Smed-sin1* at 10 days of regeneration after single or double RNAi experiments. Expression levels are relative to *gfp* RNAi injected animals (dotted line). Similar levels of downregulation for the different genes are observed in single or double RNAi experiments (P>0.05). Error bars are s.d from the mean. Asterisks indicate P<0.05 (one asterisk), P<0.01 (two asterisks and P<0.001 (three asterisks) and ns indicates “not significant” using two-tailed Student's test with equal sample variance and relative to expression in control animals. Scale bars indicate 300 µm.
(PDF)

Table S1 Terminals used for the phylogenetic analysis and their accession numbers.
(PDF)

Video S1 Cat 1 and cat 3 cells in an anterior 20 d regenerating control trunk. Only the head is shown. Anterior is to the right. *Smedwi-1* ^+^ cells (cat1) are shown in green whereas *Smed-AGAT-1* ^+^ cells are shown in magenta.
(MOV)

Video S2 Cat 1 and cat 3 cells in an anterior 20 d regenerating *Smed-smg-1(RNAi)* trunk. Only the head is shown. Anterior is defined by the magenta. *Smedwi-1* ^+^ cells (cat1) are shown in green whereas *Smed-AGAT-1* ^+^ cells are shown in magenta. Both cat1 and cat3 compartments have higher volumes than controls (see Video S1); cat1 compartment is invading cat 3 compartment, an event never happening in controls.
(MOV)

Video S3 H3P^+^ cells are present in the outgrowths. H3P^+^ cells are the green dots. The arrows indicate the presence of H3P^+^ cells in an outgrowth. Two planarians are displayed in the video.
(MOV)

Video S4 Control RNAi trunks are attracted to food and are able to eat, at 7 dR.
(AVI)

Video S5 *Smed-tor* RNAi trunks are attracted to food but are not able to eat at 7 dR. Please note anterior polarity when moving and normal movements spite of not having formed a blastema.
(AVI)

## References

[1] Aboobaker AA (2011). Planarian stem cells: a simple paradigm for regeneration.. Trends Cell Biol.

[2] Saló E (2006). The power of regeneration and the stem-cell kingdom: freshwater planarians (Platyhelminthes).. Bioessays.

[3] Baguñà J, Saló E, Auladell C (1989). Regeneration and pattern formation in planarians III. Evidence that neoblasts are totipotent stem cells and the source of blastema cells.. Development.

[4] Wagner DE, Wang IE, Reddien PW (2011). Clonogenic neoblasts are pluripotent adult stem cells that underlie planarian regeneration.. Science.

[5] Gentile L, Cebria F, Bartscherer K (2011). The planarian flatworm: an in vivo model for stem cell biology and nervous system regeneration.. Dis Model Mech.

[6] Brockes JP, Kumar A (2008). Comparative aspects of animal regeneration.. Annu Rev Cell Dev Biol.

[7] Birnbaum KD, Sanchez Alvarado A (2008). Slicing across kingdoms: regeneration in plants and animals.. Cell.

[8] Wenemoser D, Reddien PW (2010). Planarian regeneration involves distinct stem cell responses to wounds and tissue absence.. Dev Biol.

[9] Baguñà J (1976). Mitosis in the intact and regenerating planarian *Dugesia mediterranea* n. sp. II. Mitotic studies during regeneration and a possible mechanism of blastema formation.. J Exp Zool.

[10] Gonzalez-Estevez C (2009). Autophagy meets planarians.. Autophagy.

[11] Gonzalez-Estevez C, Felix DA, Rodriguez-Esteban G, Aboobaker AA (2012). Decreased neoblast progeny and increased cell death during starvation-induced planarian degrowth.. The International journal of developmental biology.

[12] Miller CM, Newmark PA (2012). An insulin-like peptide regulates size and adult stem cells in planarians.. The International journal of developmental biology.

[13] Yamashita A, Ohnishi T, Kashima I, Taya Y, Ohno S (2001). Human SMG-1, a novel phosphatidylinositol 3-kinase-related protein kinase, associates with components of the mRNA surveillance complex and is involved in the regulation of nonsense-mediated mRNA decay.. Genes Dev.

[14] Denning G, Jamieson L, Maquat LE, Thompson EA, Fields AP (2001). Cloning of a novel phosphatidylinositol kinase-related kinase: characterization of the human SMG-1 RNA surveillance protein.. J Biol Chem.

[15] Grimson A, O'Connor S, Newman CL, Anderson P (2004). SMG-1 is a phosphatidylinositol kinase-related protein kinase required for nonsense-mediated mRNA Decay in Caenorhabditis elegans.. Mol Cell Biol.

[16] Masse I, Molin L, Mouchiroud L, Vanhems P, Palladino F (2008). A novel role for the SMG-1 kinase in lifespan and oxidative stress resistance in Caenorhabditis elegans.. PLoS ONE.

[17] Brumbaugh KM, Otterness DM, Geisen C, Oliveira V, Brognard J (2004). The mRNA surveillance protein hSMG-1 functions in genotoxic stress response pathways in mammalian cells.. Mol Cell.

[18] Oliveira V, Romanow WJ, Geisen C, Otterness DM, Mercurio F (2008). A protective role for the human SMG-1 kinase against tumor necrosis factor-alpha-induced apoptosis.. J Biol Chem.

[19] Gehen SC, Staversky RJ, Bambara RA, Keng PC, O'Reilly MA (2008). hSMG-1 and ATM sequentially and independently regulate the G1 checkpoint during oxidative stress.. Oncogene.

[20] Chen RQ, Yang QK, Chen YL, Oliveira VA, Dalton WS (2009). Kinome siRNA screen identifies SMG-1 as a negative regulator of hypoxia-inducible factor-1alpha in hypoxia.. J Biol Chem.

[21] Hendzel MJ, Wei Y, Mancini MA, Van Hooser A, Ranalli T (1997). Mitosis-specific phosphorylation of histone H3 initiates primarily within pericentromeric heterochromatin during G2 and spreads in an ordered fashion coincident with mitotic chromosome condensation.. Chromosoma.

[22] Rossi L, Salvetti A, Lena A, Batistoni R, Deri P (2006). *DjPiwi-1*, a member of the PAZ-Piwi gene family, defines a subpopulation of planarian stem cells.. Dev Genes Evol.

[23] Eisenhoffer GT, Kang H, Sanchez Alvarado A (2008). Molecular analysis of stem cells and their descendants during cell turnover and regeneration in the planarian Schmidtea mediterranea.. Cell Stem Cell.

[24] Guo T, Peters AH, Newmark PA (2006). A Bruno-like gene is required for stem cell maintenance in planarians.. Dev Cell.

[25] Chai G, Ma C, Bao K, Zheng L, Wang X (2010). Complete functional segregation of planarian beta-catenin-1 and -2 in mediating Wnt signaling and cell adhesion.. J Biol Chem.

[26] Cebria F (2008). Organization of the nervous system in the model planarian Schmidtea mediterranea: an immunocytochemical study.. Neurosci Res.

[27] Cebrià F, Vispo M, Newmark P, Bueno D, Romero R (1997). Myocite differentiation and body wall muscle regeneration in the planarian *Girardia tigrina*.. Dev Gene Evol.

[28] Sakai F, Agata K, Orii H, Watanabe K (2000). Organization and regeneration ability of spontaneous supernumerary eyes in planarians -eye regeneration field and pathway selection by optic nerves.. Zoolog Sci.

[29] Baguñà J (1976). Mitosis in the intact and regenerating planarian *Dugesia mediterranea* n. sp. I. Mitotic studies during growth, feeding and starvation.. J of Exp Zool.

[30] Zoncu R, Efeyan A, Sabatini DM (2011). mTOR: from growth signal integration to cancer, diabetes and ageing.. Nat Rev Mol Cell Biol.

[31] Wullschleger S, Loewith R, Hall MN (2006). TOR signaling in growth and metabolism.. Cell.

[32] Wang X, Proud CG (2011). mTORC1 signaling: what we still don't know.. J Mol Cell Biol.

[33] Oviedo NJ, Pearson BJ, Levin M, Sanchez Alvarado A (2008). Planarian PTEN homologs regulate stem cells and regeneration through TOR signaling.. Dis Model Mech.

[34] Hara K, Maruki Y, Long X, Yoshino K, Oshiro N (2002). Raptor, a binding partner of target of rapamycin (TOR), mediates TOR action.. Cell.

[35] Zhou J, Su P, Wang L, Chen J, Zimmermann M (2009). mTOR supports long-term self-renewal and suppresses mesoderm and endoderm activities of human embryonic stem cells.. Proc Natl Acad Sci U S A.

[36] Guertin DA, Sabatini DM (2007). Defining the role of mTOR in cancer.. Cancer Cell.

[37] Lane HA, Breuleux M (2009). Optimal targeting of the mTORC1 kinase in human cancer.. Curr Opin Cell Biol.

[38] Long X, Spycher C, Han ZS, Rose AM, Muller F (2002). TOR deficiency in C. elegans causes developmental arrest and intestinal atrophy by inhibition of mRNA translation.. Curr Biol.

[39] Oldham S, Montagne J, Radimerski T, Thomas G, Hafen E (2000). Genetic and biochemical characterization of dTOR, the Drosophila homolog of the target of rapamycin.. Genes Dev.

[40] Gangloff YG, Mueller M, Dann SG, Svoboda P, Sticker M (2004). Disruption of the mouse mTOR gene leads to early postimplantation lethality and prohibits embryonic stem cell development.. Mol Cell Biol.

[41] Murakami M, Ichisaka T, Maeda M, Oshiro N, Hara K (2004). mTOR is essential for growth and proliferation in early mouse embryos and embryonic stem cells.. Mol Cell Biol.

[42] Martin PM, Sutherland AE (2001). Exogenous amino acids regulate trophectoderm differentiation in the mouse blastocyst through an mTOR-dependent pathway.. Dev Biol.

[43] Morton JP, Myant KB, Sansom OJ (2011). A FAK-PI-3K-mTOR axis is required for Wnt-Myc driven intestinal regeneration and tumorigenesis.. Cell Cycle.

[44] Gan B, Hu J, Jiang S, Liu Y, Sahin E (2010). Lkb1 regulates quiescence and metabolic homeostasis of haematopoietic stem cells.. Nature.

[45] Nakada D, Saunders TL, Morrison SJ (2010). Lkb1 regulates cell cycle and energy metabolism in haematopoietic stem cells.. Nature.

[46] Abraham RT (2004). The ATM-related kinase, hSMG-1, bridges genome and RNA surveillance pathways.. DNA Repair (Amst).

[47] Metzstein MM, Krasnow MA (2006). Functions of the nonsense-mediated mRNA decay pathway in Drosophila development.. PLoS Genet.

[48] McIlwain DR, Pan Q, Reilly PT, Elia AJ, McCracken S (2010). Smg1 is required for embryogenesis and regulates diverse genes via alternative splicing coupled to nonsense-mediated mRNA decay.. Proc Natl Acad Sci U S A.

[49] Pearson BJ, Sanchez Alvarado A (2010). A planarian p53 homolog regulates proliferation and self-renewal in adult stem cell lineages.. Development.

[50] Feldman ME, Apsel B, Uotila A, Loewith R, Knight ZA (2009). Active-site inhibitors of mTOR target rapamycin-resistant outputs of mTORC1 and mTORC2.. PLoS Biol.

[51] Thoreen CC, Kang SA, Chang JW, Liu Q, Zhang J (2009). An ATP-competitive mammalian target of rapamycin inhibitor reveals rapamycin-resistant functions of mTORC1.. J Biol Chem.

[52] Thoreen CC, Sabatini DM (2009). Rapamycin inhibits mTORC1, but not completely.. Autophagy.

[53] Choo AY, Yoon SO, Kim SG, Roux PP, Blenis J (2008). Rapamycin differentially inhibits S6Ks and 4E-BP1 to mediate cell-type-specific repression of mRNA translation.. Proc Natl Acad Sci U S A.

[54] Rouhana L, Shibata N, Nishimura O, Agata K (2010). Different requirements for conserved post-transcriptional regulators in planarian regeneration and stem cell maintenance.. Dev Biol.

[55] Robb SM, Ross E, Sanchez Alvarado A (2008). SmedGD: the Schmidtea mediterranea genome database.. Nucleic Acids Res.

[56] Blythe MJ, Kao D, Malla S, Rowsell J, Wilson R (2010). A dual platform approach to transcript discovery for the planarian Schmidtea mediterranea to establish RNAseq for stem cell and regeneration biology.. PLoS ONE.

[57] Katoh K, Misawa K, Kuma K, Miyata T (2002). MAFFT: a novel method for rapid multiple sequence alignment based on fast Fourier transform.. Nucleic Acids Res.

[58] Hall TA (1999). BioEdit: a user-friendly biological sequence alignment editor and analysis program for Windows 95/98/NT.. Nucl Acids Symp Ser.

[59] Castresana J (2000). Selection of conserved blocks from multiple alignments for their use in phylogenetic analysis.. Mol Biol Evol.

[60] Ronquist F, Huelsenbeck JP (2003). MrBayes 3: Bayesian phylogenetic inference under mixed models.. Bioinformatics.

[61] Stamatakis A (2006). RAxML-VI-HPC: maximum likelihood-based phylogenetic analyses with thousands of taxa and mixed models.. Bioinformatics.

[62] Molina MD, Neto A, Maeso I, Gomez-Skarmeta JL, Salo E (2011). Noggin and noggin-like genes control dorsoventral axis regeneration in planarians.. Curr Biol.

[63] Reddien PW, Bermange AL, Murfitt KJ, Jennings JR, Sanchez Alvarado A (2005). Identification of genes needed for regeneration, stem cell function, and tissue homeostasis by systematic gene perturbation in planaria.. Dev Cell.

[64] Pearson BJ, Eisenhoffer GT, Gurley KA, Rink JC, Miller DE (2009). Formaldehyde-based whole-mount in situ hybridization method for planarians.. Dev Dyn.

[65] Gonzalez-Estevez C, Arseni V, Thambyrajah RS, Felix DA, Aboobaker AA (2009). Diverse miRNA spatial expression patterns suggest important roles in homeostasis and regeneration in planarians.. Int J Dev Biol.

[66] Cebria F, Guo T, Jopek J, Newmark PA (2007). Regeneration and maintenance of the planarian midline is regulated by a slit orthologue.. Dev Biol.

[67] Cardona A, Fernandez J, Solana J, Romero R (2005). An in situ hybridization protocol for planarian embryos: monitoring myosin heavy chain gene expression.. Dev Genes Evol.

[68] Umesono Y, Watanabe K, Agata K (1999). Distinct structural domains in the planarian brain defined by the expression of evolutionarily conserved homeobox genes.. Dev Genes Evol.

[69] Gonzalez-Estevez C, Felix DA, Aboobaker AA, Salo E (2007). Gtdap-1 promotes autophagy and is required for planarian remodeling during regeneration and starvation.. Proc Natl Acad Sci U S A.

[70] Cebria F, Newmark PA (2005). Planarian homologs of netrin and netrin receptor are required for proper regeneration of the central nervous system and the maintenance of nervous system architecture.. Development.

[71] Hayashi T, Asami M, Higuchi S, Shibata N, Agata K (2006). Isolation of planarian X-ray-sensitive stem cells by fluorescence-activated cell sorting.. Dev Growth Differ.

[72] Reddien PW, Oviedo NJ, Jennings JR, Jenkin JC, Sanchez Alvarado A (2005). SMEDWI-2 is a PIWI-like protein that regulates planarian stem cells.. Science.

