# Supplementary figures and images for: SMG-1 and mTORC1 Act Antagonistically to Regulate Response to Injury and Growth in Planarians
Source: PLoS Genet. 2012 Mar 29;8(3):e1002619. doi: 10.1371/journal.pgen.1002619 (PMC3315482; doi:10.1371/journal.pgen.1002619)

A

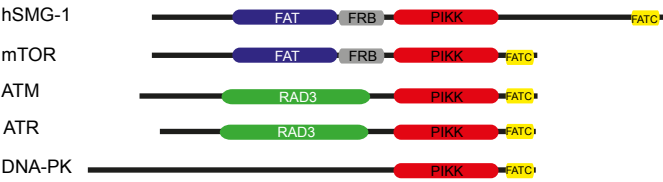

B

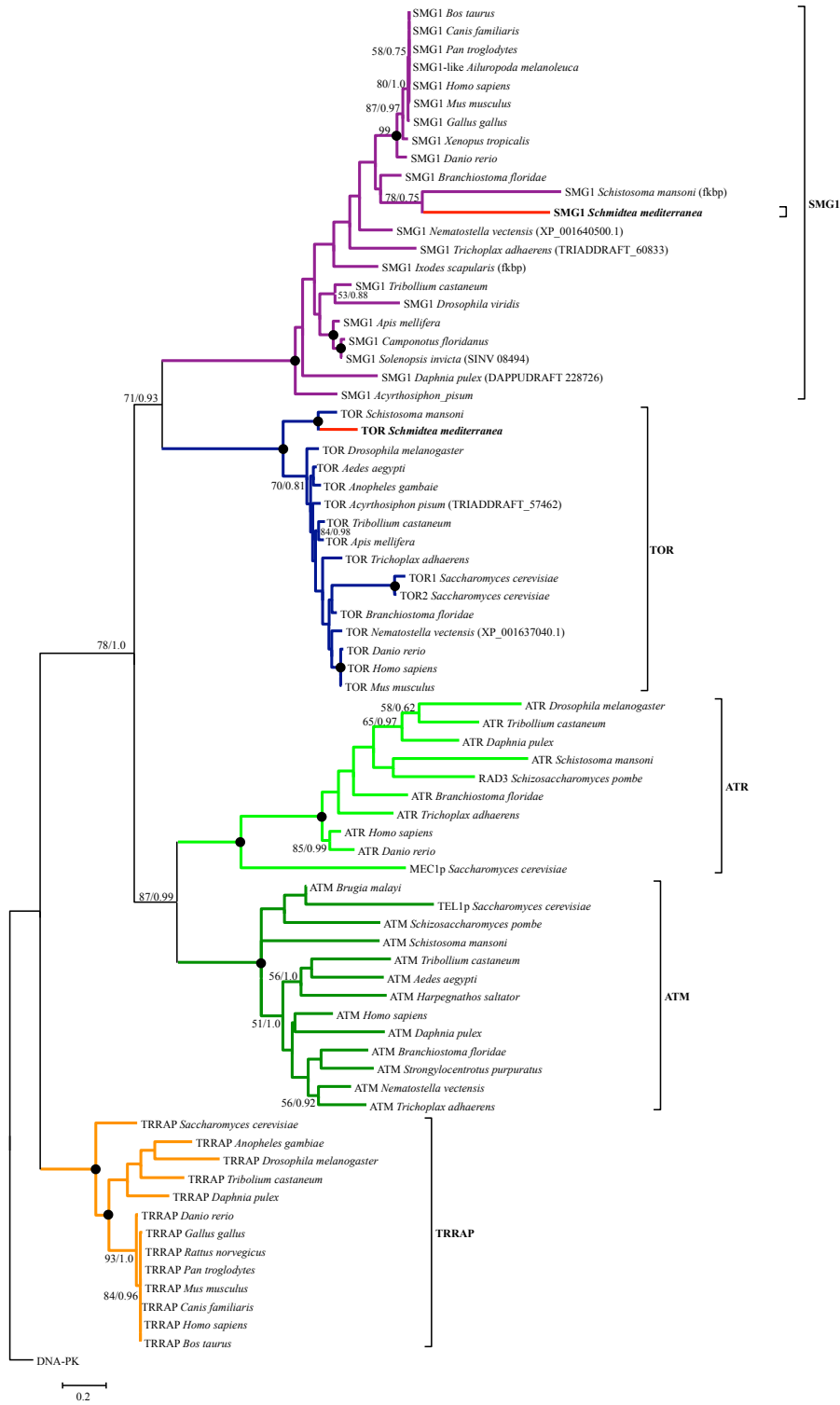

Supplement: Figure S1 — SMED-SMG-1, the homolog of hSMG-1, and SMED-TOR, the homolog of mTOR, are members of the PIKK family of proteins. A. Schematic drawing of the domains present in all the members of the PIKK family of proteins. The proteins displayed are the ones present in humans. B. Maximum Likelihood phylogenetic tree of the PIKK+FATC domain in PIKK proteins. Phylogeny inferred with RAxML (GTR+Γ+I), numbers correspond respectively to Bootstrap supports and Bayesian inference Posterior Probabilities. A black dot indicates a clade with Bootstrap support superior to 95% and Bayesian Posterior Probabilities (PP) values of 1,0. Values under 50% or 0.5 are not indicated. The scale bar indicates the number of changes per site. For accession numbers corresponding to each terminal see Table S1. (PDF) [file pgen.1002619.s001.pdf]

### E value vs *C. elegans*

4 e-93

## FRB

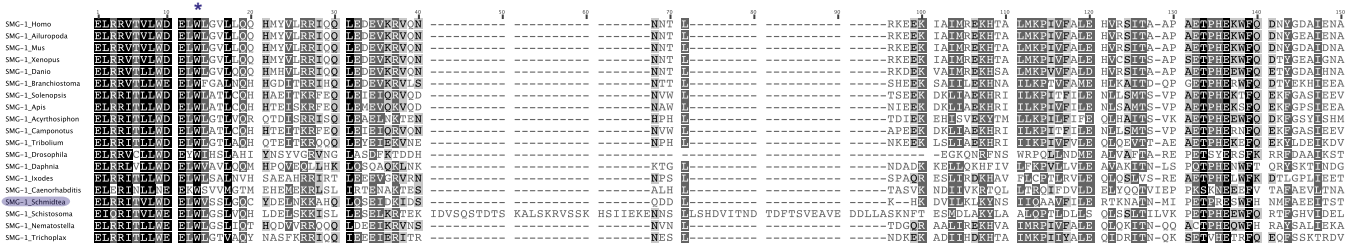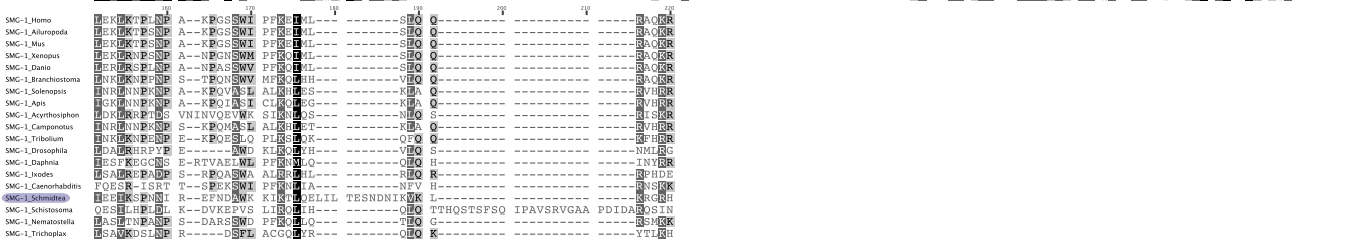

## PIKK

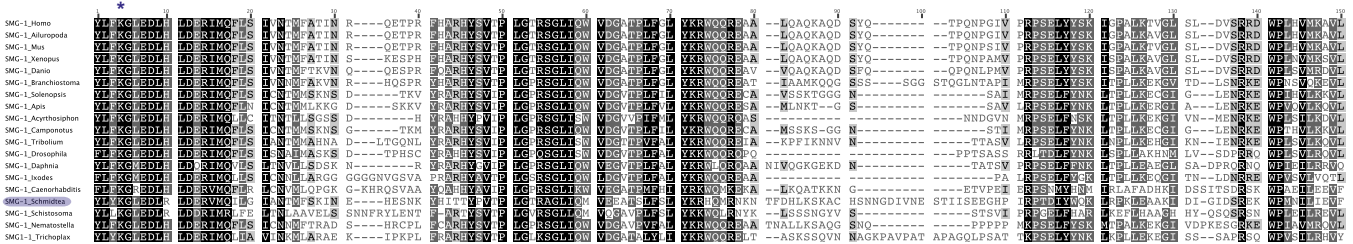

## FATC

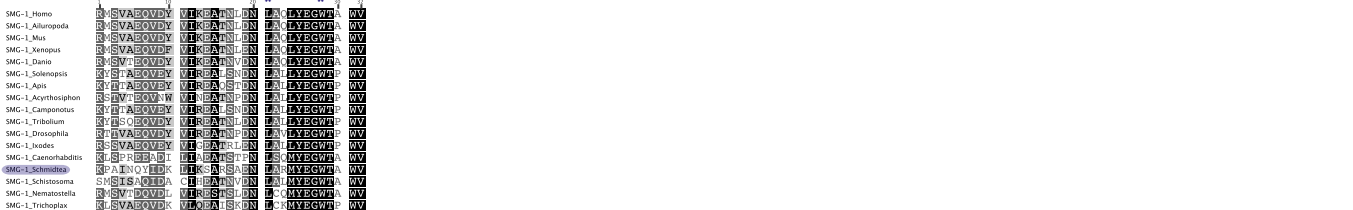

Supplement: Figure S2 — Smed-smg-1 is a bona fide SMG-1. A. Schematic illustration of the domains present on SMED-SMG-1 compared to hSMG-1. B. Multiple alignment (Blosum 62) for the FRB, PIKK and FATC domains of SMG-1 from several organisms. The critical tryptophan residue required for mTOR kinase activity [14] is conserved in SMG-1 indicated with an asterisk in the FRB alignment. hSMG-1 contains several highly conserved motifs found in all PIK-related kinases [13], [14]. A conserved ATP-binding site indicated by the asterisk in the PIKK domain is conserved in all the SMG-1 proteins from the different organisms displayed and motif I and motif II sites within the catalytic domain indicated by the blue lines are also conserved in all the SMG-1 proteins. Residues, whose substitution decreases hSMG-1 kinase activity, are labelled with an asterisk in the FATC alignment and are conserved in all the organisms displayed. (PDF) [file pgen.1002619.s002.pdf]

**A**

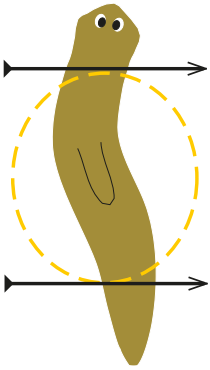**B**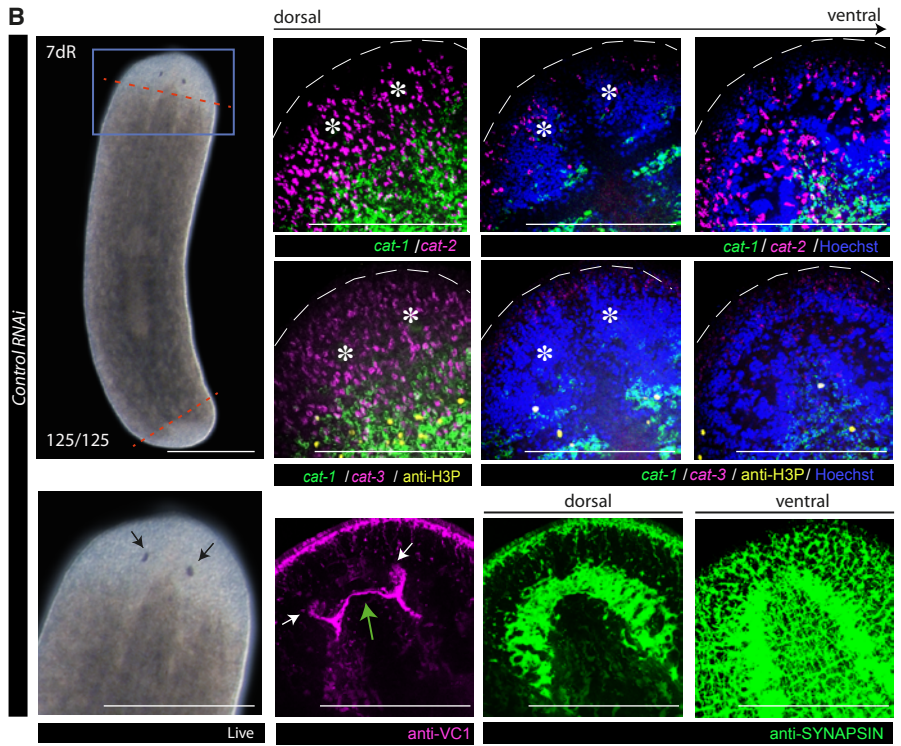

*Smed-smg-1 RNAi*

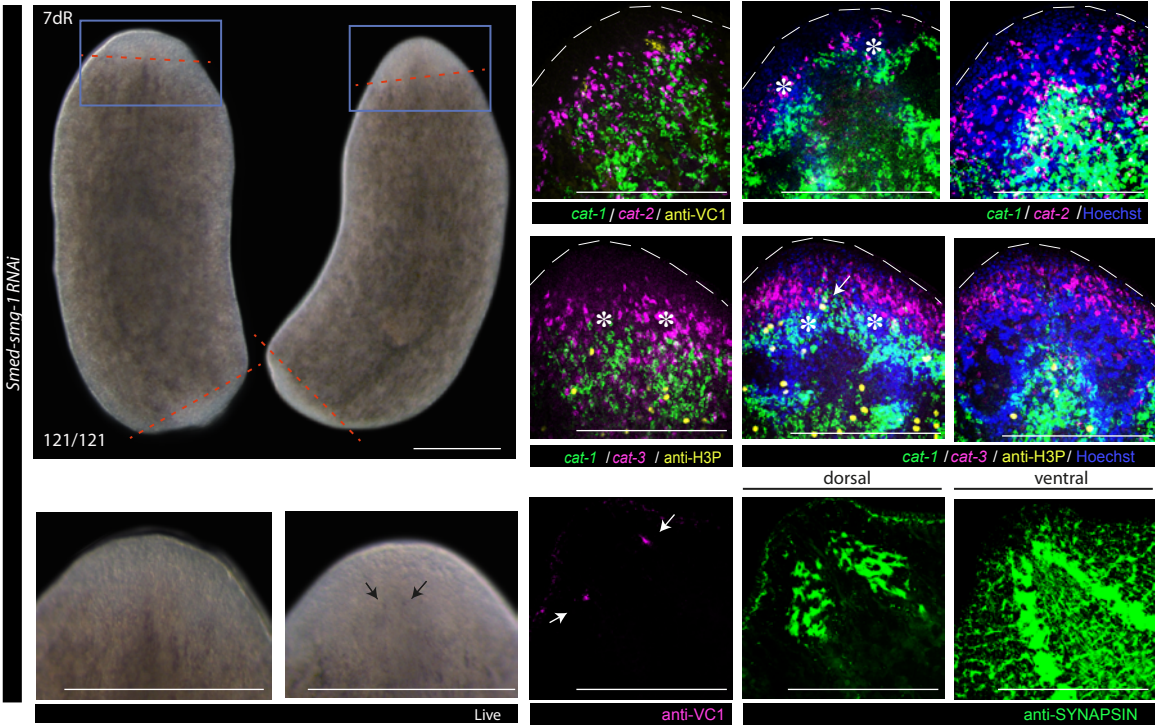

Supplement: Figure S3 — Smed-smg-1 is required for correct blastema growth and to restrict the category 1 compartment during regeneration. A. The cartoon shows the levels of transverse amputation performed in the experiment (arrows) and highlights the trunk piece (dotted yellow circle), which was kept to follow anterior and posterior regeneration in all the experiments. B. Panels show 7 dR trunks. Dotted red lines define the blastemas and the blue square shows the area displayed in the fluorescent panels. Control animals show an anterior blastema with a couple of eyes, where the pigmentary cup can be seen in the live images (black arrows) and the photosentitive cells and optic chiasm in anti-VC1 staining (white arrows and green arrow, respectively). Smed-smg-1(RNAi) animals show varying sizes of blastemas with a low degree of eye differentiation displayed in the live images (black arrows). The anti-VC1 staining shows the presence of underdeveloped photosensitive cells (white arrows) and no optic chiasma. Panels show maximum projections of the dorsal-most confocal sections, the medial-most confocal sections and the ventral-most confocal sections of anterior regeneration in 7 dR trunks. Asterisks indicate the position of the eyes. Panels show the distribution of neoblasts (cat-1), neoblast early progeny (cat-2) and neoblast late progeny (cat-3) markers (n≥4). The white arrow indicates the presence of cat 1 and H3P+ cells in the cat-2 and cat-3 compartments in front of the eyes in Smed-smg-1(RNAi) animals. Lower panels show a very undeveloped brain as seen by synapsin staining (dorsal-most maximum confocal projection) similar to the phenotype at 20 dR (Figure 2 in main manuscript). Scale bars indicate 300 µm. (PDF) [file pgen.1002619.s003.pdf]

**A**

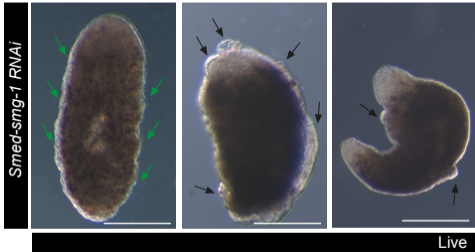

**B**

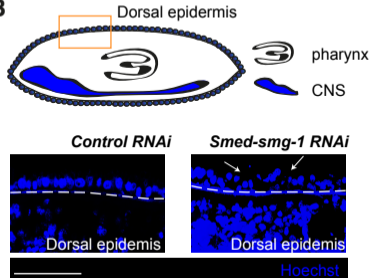

Supplement: Figure S4 — During regeneration, Smed-smg-1(RNAi) animals with abnormal blastemas progressed to form outgrowths and die. A. Panels show some examples of planarians at 30–35 dR presenting hyperplasia and outgrowths. Green arrows indicate epidermal hyperplasia and black arrows indicate outgrowths. Scale bars indicate 300 µm. B. The cartoon represents a sagittal paraffin section. The orange square shows the area of the section displayed in the fluorescent panels. Panels are confocal maximum projections of slices performed on 10 µm paraffin sections. White arrows indicate the multilayered epidermis in Smed-smg-1(RNAi) animals (n = 10/10 planarians). Scale bar indicates 50 µm. (PDF) [file pgen.1002619.s004.pdf]

# González-Estévez\_Fig. S5

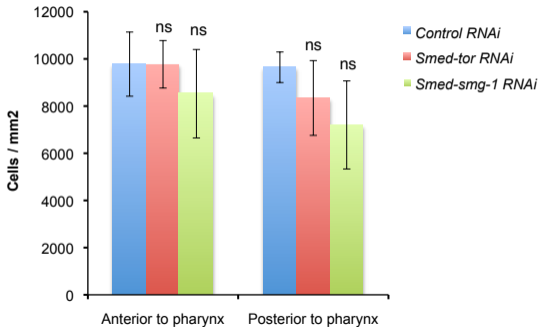

Supplement: Figure S5 — Smed-smg-1(RNAi) and Smed-tor(RNAi) animals show similar number of Smedwi-1 + cells before amputation to control RNAi animals. Smedwi-1+ cells quantification in 0.035 mm2 equivalent regions of the animals, anterior and posterior to the pharynx, prior to amputation in Smed-smg-1 and Smed-tor RNAi animals. Error bars are s.d from the mean. No significant differences P>0.05 (ns) were observed in relation to control RNAi animals using two-tailed Student's test with equal sample variance. ≥5 planarians per time point. (PDF) [file pgen.1002619.s005.pdf]

# González-Estévez\_Fig. S6

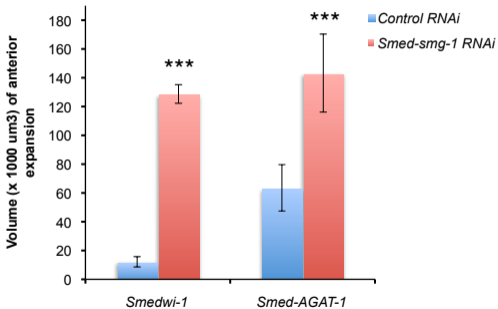

Supplement: Figure S6 — Smed-smg-1 RNAi animals show anterior expansion of cat-1 and cat-3 compartments. Volume in µm3 of cat-1 and cat-2 compartments in Smed-smg-1 RNAi compared to control RNAi animals quantified from the region of the eyes until the tip of the head. Error bars are s.d from the mean and three asterisks indicate P<0.001 using two-tailed Student's test with equal sample variance relative to control RNAi animals. (PDF) [file pgen.1002619.s006.pdf]

20dR posterior blastemas

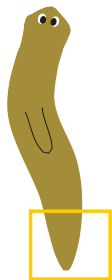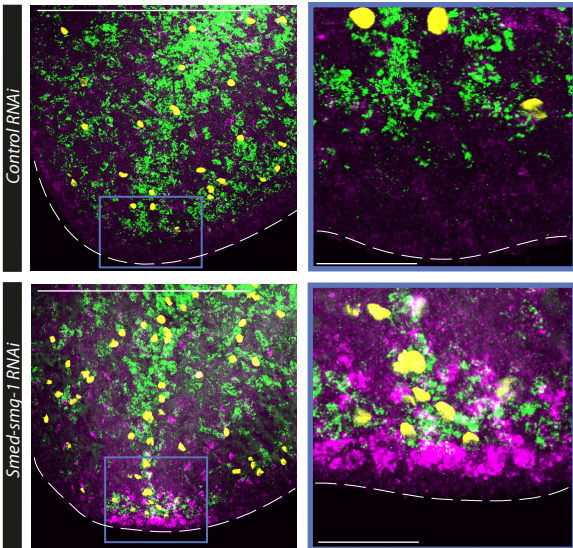

Supplement: Figure S7 — Smed-smg-1(RNAi) animals show a posterior-dorsal expansion of the neoblast compartment at posterior-facing blastemas and an accumulation of late neoblast progeny at the tip of the blastema. The yellow square on the cartoon shows the area displayed in the fluorescent panels. Panels show maximum projections of 20 d posterior regenerating trunks. Panels show the distribution of neoblasts (cat-1), H3P+ cells, and neoblast late progeny (cat-3) markers (n = 6/6). The blue box indicates the area of high magnification. Scale bars indicate 300 µm and 50 µm in the high magnifications. (PDF) [file pgen.1002619.s007.pdf]

**A**

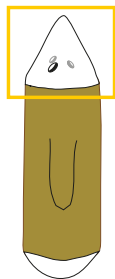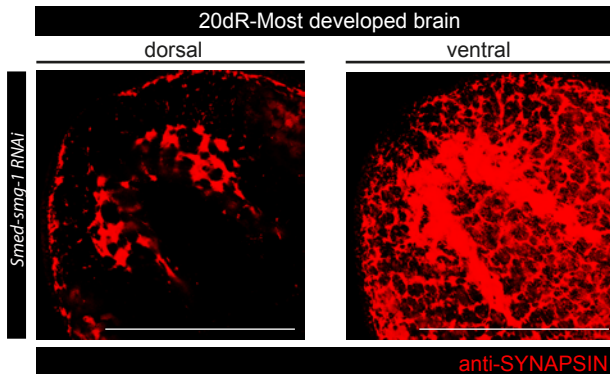

**B**

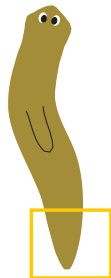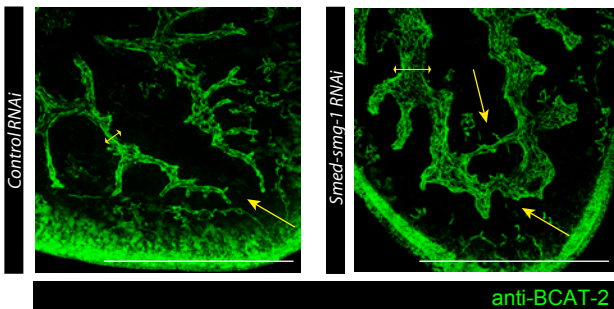

Supplement: Figure S8 — Smed-smg-1(RNAi) animals display differentiation problems during regeneration. A. The yellow square on the cartoon shows the area displayed in the fluorescent panels. Panels show maximum projections of the dorsal-most and the ventral-most confocal sections for the most developed brain observed at 20 dR planarians (stained with anti-SYNAPSIN). Control planarians for these images are seen in Figure 2E in main manuscript. B. The yellow square on the cartoon shows the area displayed in the fluorescent panels. Panels show maximum projections of 20 days posterior regenerating trunks. Anti-BCAT-2 shows the epithelia of the gut. Arrows indicate that the end of the posterior gut branches display anastomoses in Smed-smg-1(RNAi) animals but are not fused in controls (n = 6/6 versus n = 0/6 in controls). Double arrows show that the posterior gut branches are thicker in Smed-smg-1(RNAi) than controls (n = 6/6). It was not possible to determine from this experiment if the higher thickness is due to an increase in cell number or an increase in cell size. Scale bars indicate 300 µm. (PDF) [file pgen.1002619.s008.pdf]

A

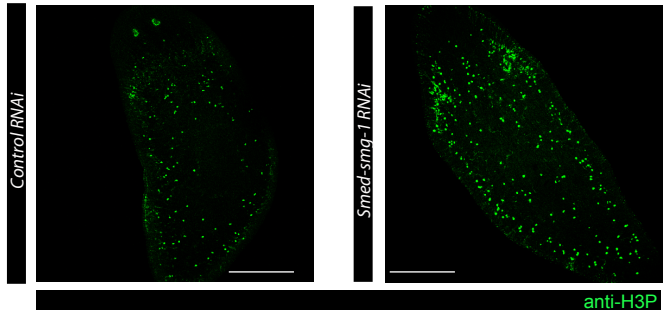

B

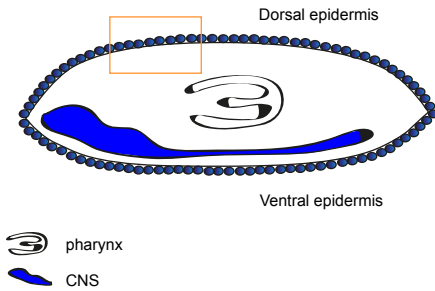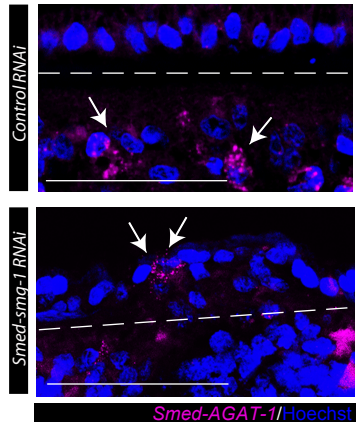

C

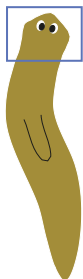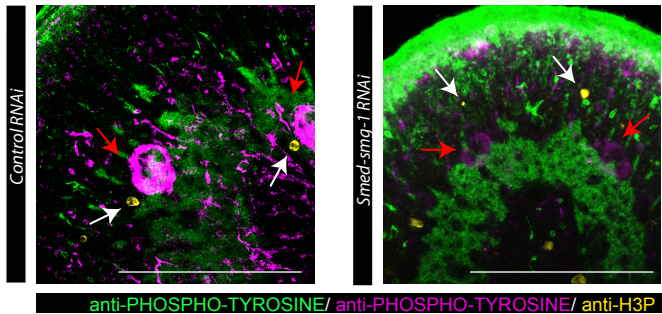

Supplement: Figure S9 — Homeostatic Smed-smg-1(RNAi) animals show hyper-proliferation, mitoses in front of the eyes with late neoblast progeny rarely observed in outgrowths. A. Representative images showing H3P staining at 9 days after the last injection. Scale bars indicate 300 µm. B. The cartoon shows a sagittal paraffin section. The orange square shows the region represented in the panels. The arrows indicate Smed-AGAT-1 + cells (n = 1/4 planarians). C. The cartoon indicates the area showed in the panels. Panels are confocal projections of anti-phospho-tyrosine immunofluorescence, pseudocolored according to the depth of the focal plane: dorsal-most sections are shown in magenta; deeper sections in green. Smed-smg-1(RNAi) animals display mitosis (white arrows) in front of the eyes (red arrows) (n = 3/7). Scale bars indicate 150 µm. Scale bars indicate 50 µm. (PDF) [file pgen.1002619.s009.pdf]

**A**
*Smed-Ist8*

E value vs Human

 $4 \times 10^{-78}$ 
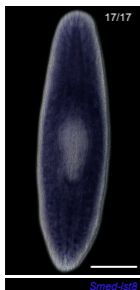
**B**
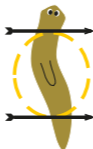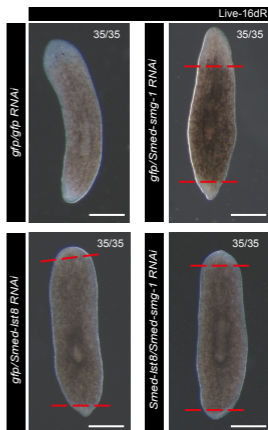
**C**
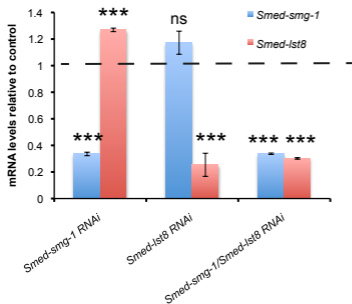

Supplement: Figure S12 — Smed-lst8 is a bona fide LST8, is broadly expressed in the whole planarian body and RNAi experiments show a very weak phenotype however similar to the ones obtained for Smed-tor and Smed-raptor. A. Smed-lst8 is broadly expressed in the planarian body (n = 30/30 in three independent experiments). B. The cartoon shows the levels of transverse amputation performed in the experiment (arrows) and highlights the trunk piece (dotted red circle), which was kept to follow anterior and posterior regeneration in all the experiments. Panels show 16 dR trunks. Dotted red lines define the blastemas. Smed-lst8 RNAi planarians show a reduced blastema compared to controls. Double Smed-smg-1/Smed-lst8 RNAi experiments show Smed-lst8 phenotype. C. Relative expression of Smed-smg-1 and Smed-lst8 at 10 days of regeneration after single or double RNAi experiments. Expression levels are relative to gfp RNAi injected animals (dotted line). Similar levels of downregulation for the different genes are observed in single or double RNAi experiments (P>0.05). Error bars are s.d from the mean. Asterisks indicate P<0.001 (three asterisks) and ns indicates “not significant” using two-tailed Student's test with equal sample variance and relative to expression in control animals. Scale bars indicate 300 µm. (PDF) [file pgen.1002619.s012.pdf]

**A***Smed-riCTOR*

E value vs Human  
4 e -42

*Smed-sin1*

E value vs Human  
4 e -17

**B**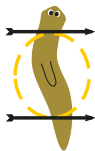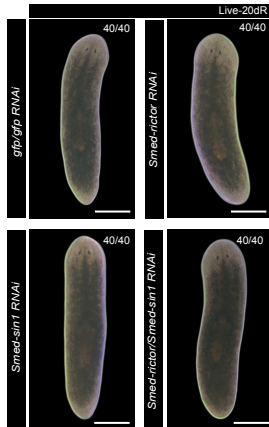**C**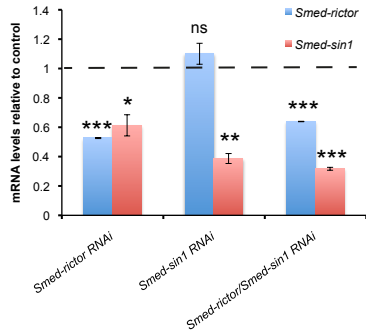

Supplement: Figure S13 — Smed-rictor and Smed-sin1 are bona fide RICTOR and SIN1, respectively. RNAi experiments of both genes either alone or combined showed no phenotype. A. E-value of the genes respect to the human homolog. B. The cartoon shows the levels of transverse amputation performed in the experiment (arrows) and highlights the trunk piece (dotted red circle), which was kept to follow anterior and posterior regeneration in all the experiments. Panels show 20 dR trunks. Dotted red lines define the blastemas. Smed-rictor, Smed-sin1 or Smed-rictor/Smed-sin1 RNAi did not show any phenotype even after three rounds of RNAi and regeneration. C. Relative expression of Smed-rictor and Smed-sin1 at 10 days of regeneration after single or double RNAi experiments. Expression levels are relative to gfp RNAi injected animals (dotted line). Similar levels of downregulation for the different genes are observed in single or double RNAi experiments (P>0.05). Error bars are s.d from the mean. Asterisks indicate P<0.05 (one asterisk), P<0.01 (two asterisks and P<0.001 (three asterisks) and ns indicates “not significant” using two-tailed Student's test with equal sample variance and relative to expression in control animals. Scale bars indicate 300 µm. (PDF) [file pgen.1002619.s013.pdf]
