# Supplementary material for: SMG-1 and mTORC1 Act Antagonistically to Regulate Response to Injury and Growth in Planarians
Source: PLoS Genet. 2012 Mar 29;8(3):e1002619. doi: 10.1371/journal.pgen.1002619 (PMC3315482; doi:10.1371/journal.pgen.1002619)

González-Estévez_Fig. S10

A

RNAi phenotype	33d-*GFP*	9d-*Smed-smg-1*	22d-*Smed-smg-1*	33d-*Smed-smg-1*
Normal	100% (84/84)	20.24% (17/84)		
Hyperplasia		34.52% (29/84)	7.14% (6/84)	
Hyperplasia and outgrowths		35.71% (30/84)	61.90% (52/84)	
Dead after outgrowths		9.52% (8/84)	30.95% (26/84)	100% (87/87)

B

Trunks 25dR	*Control RNAi*	*Smed-smg-1 RNAi*
Normal	100% (75/75)	8% (6/75)
Abnormal unpigmented blastemas with or without hyperplasia		38.67% (29/75)
Abnormal unpigmented blastemas, hyperplasia and/or outgrowths		9.33% (7/75)
Dead after outgrowths		44% (33/75)

C

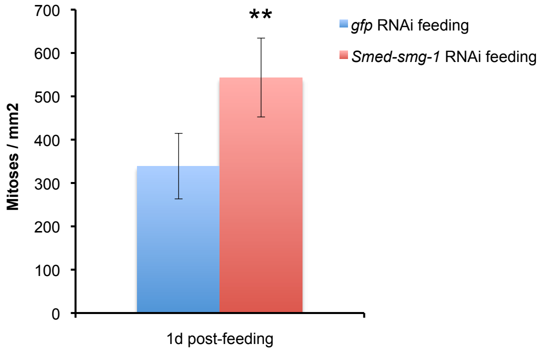

Supplement: Figure S10 — Smed-smg-1 restricts the mitotic response to feeding. A, B. Tables show Smed-smg-1(RNAi) feeding phenotype progression compared to controls. d are days after the last injection. A. Homeostasis phenotypes. B. Phenotypes in trunks at 25 d of regeneration. C. Number of H3P positive cells per mm2 in control and Smed-smg-1 RNAi planarians 1 d after feeding the corresponding dsRNAs. Error bars are s.d from the mean, the asterisks indicate P<0.01 using two-tailed Student's test with equal sample variance relative to the controls. n≥5. (PDF) [file pgen.1002619.s010.pdf]
