# Supplementary material for: SMG-1 and mTORC1 Act Antagonistically to Regulate Response to Injury and Growth in Planarians
Source: PLoS Genet. 2012 Mar 29;8(3):e1002619. doi: 10.1371/journal.pgen.1002619 (PMC3315482; doi:10.1371/journal.pgen.1002619)
Supplement: Figure S11 — SMED-TOR and SMED-RAPTOR conserved domains. A. Schematic illustration of the domains present on SMED-TOR compared to human mTOR. B. Schematic illustration of the domains present on SMED-RAPTOR compared to human RAPTOR. RNC domain accounts for RAPTOR N-terminal conserved domain. (PDF) [file pgen.1002619.s011.pdf]

González-Estévez_Fig. S11

A

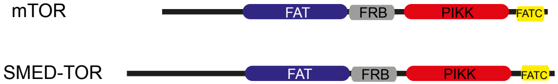

E value vs Human

3 e 0.0

E value vs *D. melanogaster*

2 e-178

B

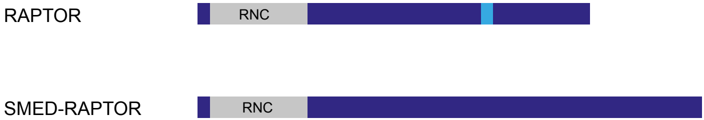

E value vs Human

4 e-118

E value vs *D. melanogaster*

3 e-111
