# Supplementary material for: SMG-1 and mTORC1 Act Antagonistically to Regulate Response to Injury and Growth in Planarians
Source: PLoS Genet. 2012 Mar 29;8(3):e1002619. doi: 10.1371/journal.pgen.1002619 (PMC3315482; doi:10.1371/journal.pgen.1002619)
Supplement: Table S1 — Terminals used for the phylogenetic analysis and their accession numbers. (PDF) [file pgen.1002619.s014.pdf]

González-Estévez_Table S1

**Terminals**	**Accession Numbers**
SMG-1 *Homo sapiens*	NP_055907.3
SMG-1 *Pan troglodytes*	XP_001152224.1
SMG-1 *Canis familiaris*	XP_851552.1
SMG-1 *Bos taurus*	XP_603490.3
SMG-1 *Mus musculus*	NP_001026984.1
SMG-1 *Gallus gallus*	XP_414907.2
SMG-1 *Danio rerio*	NP_001073513.2
SMG-1 *Ailuropoda melanoleuca*	XP_002920515.1
SMG-1 *Xenopus tropicalis*	XP_002932267.1
SMG-1 *Triboluim castaneum*	XP_969532.1
SMG-1 *Nematostella vectensis*	XP_001640500.1
SMG-1 *Branchiostoma floridae*	XP_002607176.1
SMG-1 *Camponotus floridanus*	EFN74897.1
SMG-1 *Solenopsis invicta*	EFZ20104.1
SMG-1 *Apis mellifera*	XP_001122895.1
SMG-1 *Acyrtosiphon pisum*	XP_001947123.1
SMG-1 *Drosophila virilis*	XP_002057072.1
SMG-1 *Trichoplax adhaerens*	XP 002116869.1
SMG-1 *Daphnia pulex*	EFX69271.1
SMG-1 *Schistosoma mansoni*	XP 002575156.1
SMG-1 *Ixodes scapularis*	XP 002414133.1
TOR *Homo sapiens*	NP 004949.1
TOR *Mus musculus*	NP 064393.1
TOR *Danio rerio*	NP 001070679.2
TOR *Drosophila melanogaster*	NP 524891.1
TOR *Anopheles gambiae*	XP 317619.2
TOR *Branchiostoma floridae*	EEA56493.1
TOR *Apis mellifera*	XP 625130.1
TOR *Tribolium castaneum*	XP 971819.1
TOR *Acyrthosiphon pisum*	XP 001948118.1
TOR *Trichoplax adhaerens*	XP 002113370.1
TOR *Aedes aegypti*	XP 001650255.1
TOR *Nematostella vectensis*	XP 001637040.1
TOR1 *Saccharomyces cerevisiae*	CAA52849.1
TOR2 *Saccharomyces cerevisiae*	NP 012719.2
TOR *Schistosoma mansoni*	XP 002571630.1
ATR *Homo sapiens*	NP 001175.2
ATR *Danio rerio*	XP 696163.4
ATR *Schistosoma mansoni*	XP 002578622.1
ATR *Trichoplax adhaerens*	XP 002110572.1
ATR *Daphnia pulex*	EFX77801.1
ATR *Branchiostoma floridae*	XP 002594547.1
ATR *Tribolium castaneum*	EFA09227.1
ATR *Drosophila melanogaster*	AAC46881.1
RAD3 *Schizosaccharomyces pombe*	NP 595357.1
MEC1P *Saccharomyces cerevisiae*	NP 009694.1
ATM *Homo sapiens*	NP 000042.3
Tel1p *Saccharomyces cerevisiae*	AAA69802.1
ATM *Brugia malayi*	XP 001899536.1
ATM *Harpegnathos saltator*	EFN88218.1
ATM *Aedes aegypti*	XP 001649972.1
ATM *Branchiostoma floridae*	ACG68443.1
ATM *Strongylocentrotus purpuratus*	NP 001107666.1
ATM *Daphnia pulex*	EFX88021.1
ATM *Nematostella vectensis*	XP 001628467.1
ATM *Trichoplax adhaerens*	XP 002118267.1
ATM *Tribolium castaneum*	EFA11787.1
ATM *Schizosaccharomyces pombe*	NP 588126.1
ATM *Schistosoma mansoni*	XP 002577847
TRRAP *Homo sapiens*	NP 003487.1
TRRAP *Pan troglodytes*	XP 001136894.1
TRRAP *Canis familiaris*	XP 860949.1
TRRAP *Bos taurus*	XP 583735.3
TRRAP *Mus musculus*	NP 001074831.1
TRRAP *Rattus norvegicus*	XP 213706.4
TRRAP *Gallus gallus*	XP 414752.2
TRRAP *Danio rerio*	XP 001919276.2
TRRAP *Drosophila melanogaster*	NP 001097192.1
TRRAP *Anopheles gambiae*	XP 556172.2
TRRAP *Saccharomyces cerevisiae*	NP 011967.1
TRRAP *Daphnia pulex*	EFX90201.1
TRRAP *Tribolium castaneum*	EFA05277.1
DNA-PK *Homo sapiens*	P78527.3
